# Quantifying the Accuracy, Uncertainty, and Sensitivity of Soil Geochemical Multisurface Models

**DOI:** 10.1021/acs.est.4c04812

**Published:** 2025-03-05

**Authors:** Wietse Wiersma, Elise Van Eynde, Rob N. J. Comans, Jan E. Groenenberg

**Affiliations:** † Soil Chemistry Group, Wageningen University & Research, 6708 PB Wageningen, The Netherlands; ‡ Soil Biology Group, Wageningen University & Research, 6708 PB Wageningen, The Netherlands; § European Commission, Joint Research Centre (JRC), 21027 Ispra, Italy

**Keywords:** affinity nonideality, assemblage model, environmental protection, generic adsorption parameters, heavy metals

## Abstract

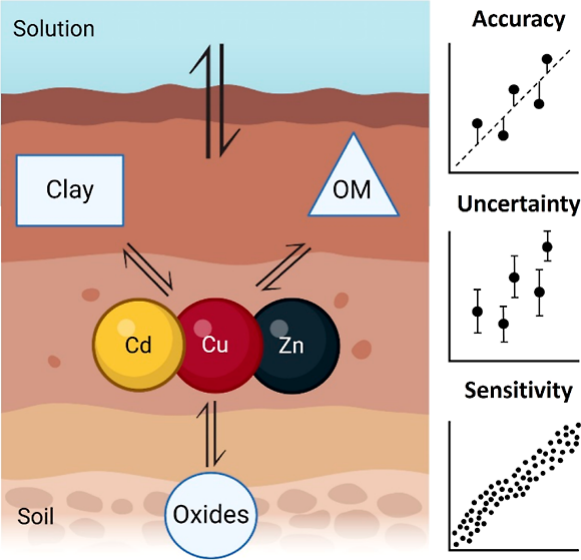

Geochemical multisurface models and their generic parameters for the solid-solution partitioning and speciation of metals have been used for decades. For soils the collective uncertainty and sensitivity of model parameters and soil-specific reactive surface properties has been insufficiently evaluated. We used statistical tools and data of diverse soils to quantify for Cd, Cu and Zn the uncertainty of model parameters and input values of the nonideal competitive adsorption (NICA)-Donnan model for organic matter (OM) coupled with the generalized two-layer model for metal-oxides. Subsequently, we quantified the uncertainty of speciation predictions and the sensitivity to model parameters and input values. Importantly, we established new generic NICA-Donnan parameters that substantially improved model accuracy, especially for Zn. Uncertainties generally followed Cu < Cd < Zn. With OM being the major binding surface across most soils, the affinity parameters (log *K*_*i*_) were most influential. Compared to a “best-case” scenario with all relevant soil properties measured, a “simplified” scenario with assumptions about OM fractionation and metal-oxide specific surface area could be employed with a negligible effect on model accuracy and uncertainty. Our study provides a reference work with quantitative measures of model performance, which facilitates broader adoption of mechanistic multisurface models in addressing environmental challenges.

## Abstract 2

Evaluating the performance of models that predict the fate of heavy metals in soils is essential for accurate and precise environmental protection criteria.

## Introduction

Geochemical models are powerful tools to capture and supplement our understanding of the behavior of contaminants, heavy metals and nutrients in the environment, including aquatic systems, sediments, and soils. For soils, such models are employed to understand the availability, mobility and transport of elements by quantifying the solid-solution partitioning and speciation.^2^ Especially potent are multisurface models (MSMs), which combine submodels for each soil reactive surface and account for element-specific binding to pH-dependent variable-charge surfaces, and for unspecific binding through electrostatic interactions.

Geochemical MSMs have the potential to contribute to solutions for soil-related societal challenges, facilitated by the development of generic adsorption parameters.^1,3^ Examples include establishing site-specific environmental protection criteria for sustainable landfilling in The Netherlands,^4^ or supporting nation-wide policies to mitigate Cd uptake by rice in China.^5^ To support the adoption of MSMs for such purposes, it is essential to comprehensively evaluate MSM performance in terms of accuracy, uncertainty and sensitivity. Sources of MSM uncertainty include (i) conceptual uncertainty related to model formulation, (ii) uncertainty in model parameters and (iii) uncertainty in soil-specific input values.^6^ Here, we focus on uncertainty in model parameters and input values related to the adsorption of metals to metal-oxides and organic matter (OM).^2^ For metal-oxides, we use the common two-site diffuse double layer model of Dzombak and Morel,^3^ i.e., the generalized two-layer model (GTLM). For OM, we used the advanced nonideal competitive adsorption model, coupled with a Donnan model (NICA-Donnan) for electrostatic effects.^7,8^

Both the GTLM and NICA-Donnan model recognize heterogeneity in the metal binding affinity and capacity of reactive surfaces. Metal-oxide adsorption in the GTLM is described with two types of binding sites (weak and strong). Acid–base titration and adsorption experiments used to derive binding constants are generally performed with well-defined synthetic metal-oxides, limiting parameter uncertainty. However, the specific surface area (SSA) has been shown to vary between soils up to an order of magnitude.^9^ The NICA-Donnan model considers humic acid (HA) and fulvic acid (FA) and describes binding to two types of functional groups (low-affinity carboxylic and high-affinity phenolic), with the average stoichiometry of the reaction captured by a nonideality parameter.^8^ Humic substances isolated from soils have been found to show substantial variability in their binding properties.^1,10^ An analytical challenge in determining NICA-Donnan parameters is to cover a sufficiently wide range of pH values and free metal concentrations. The latter has been enabled by recent techniques such as the Donnan membrane technique (DMT) and absence of gradients and Nernstian equilibrium stripping (AGNES).^11,12^

Previously, uncertainty and sensitivity analyses have been performed for metal speciation in surface water^13^ and specifically in solutions containing organic matter modeled with the NICA-Donnan model^6^ or the WHAM/model VI.^14^ The latter two studies concluded that prediction uncertainty was related to both generic and element-dependent parameters. Uncertainty for metals with a relatively low affinity for organic matter (e.g., Cd) may originate mainly from uncertainty in the fraction of OM that is reactive (e.g., humic or fulvic acid), whereas for elements with a high affinity (e.g., Cu) the uncertainty may come mainly from uncertainty in the binding affinity.^6^

**Figure 1:**
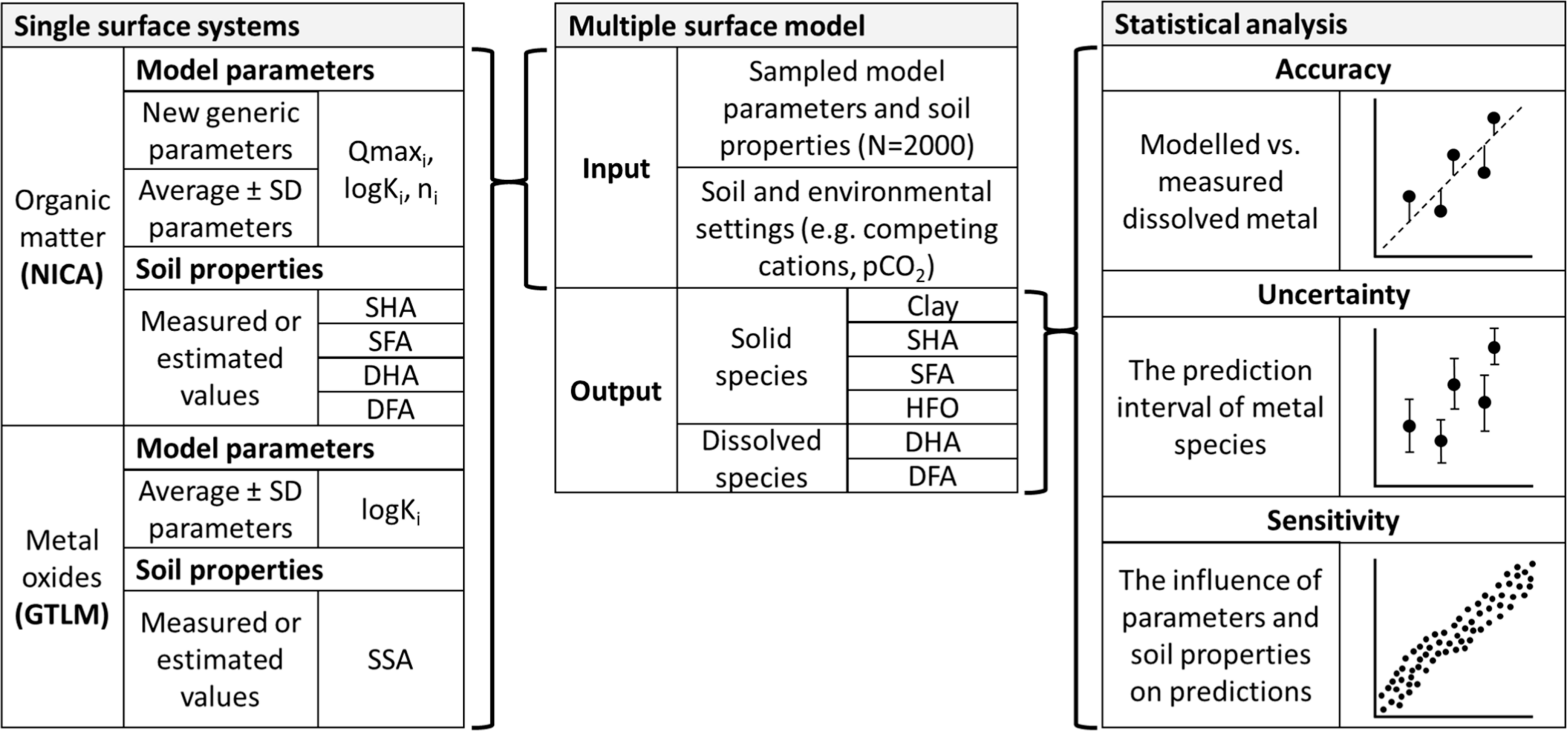
Schematic overview of the methodology followed in this study. The difference between the local and global scenario is whether soil properties were measured or estimated, respectively.

We focus in this study on the trace metals cadmium, copper and zinc. Copper has the largest availability of adsorption data sets and is generally well-modeled.^1^ Cadmium is a potentially carcinogenic metal that is regulated in food products, whereas zinc is a chemical analogue of cadmium yet is an essential micronutrient.^15^ With the emergence of new data, we derived new generic parameters for the NICA model, updating the seminal Milne et al.^1^ values. Next, we compiled a database of 24 highly diverse soils from around the world and quantified (i) the uncertainty in NICA-Donnan and GTLM model parameters and soil-specific input values, (ii) the resulting overall accuracy and uncertainty of MSM predictions, (iii) the sensitivity of MSM predictions to model parameters and input values and (iv) the impact of soil properties on the observed uncertainty and sensitivity (Figure 1).

## Materials and Methods

### Soil Selection and Characterization

We selected 24 topsoil samples (0–20 cm) from a range of available samples that were stored at Wageningen University & Research, The Netherlands. These soils were (partially) analyzed in previous studies, and missing data were determined within the present study. The soils originated from Rwanda and Burundi,^16,17^ China,^18^ The Netherlands,^19^ Ireland,^20^ Colombia and Ecuador (Supporting Information SI.1). Soils are referenced by their country of origin code followed by the code used in the original publication. Clay content was determined by the sieve and pipet method or by laser diffraction. The contents of crystalline and amorphous iron and aluminum oxides were calculated based on dithionite and ammonium oxalate extractions, as described in SI.1. Crystalline and amorphous Al and Fe oxides were added together,^21^ corrected for the difference in specific surface area, to be modeled with the GTLM as hydrous ferric oxide (HFO). The reactive surface area (RSA) was determined with the PO_4_ probe-ion method with ferrihydrite as the model oxide.^9^ Subsequently, the specific surface area (SSA) was calculated as explained by Mendez et al.^16^ This method was developed for the CD-MUSIC model, indicating that the SSA could have been different with the GTLM. We acknowledge this discrepancy as a limitation of our model framework, yet our results showed that variation in the SSA had a negligible influence on prediction uncertainty and sensitivity (see below).

Solid and dissolved [in 10 mM Ca(NO_3_)_2_] organic matter were fractionated into humic acid (HA) and fulvic acid (FA).^22^ OM was assumed to contain 50% carbon. Because FA and hydrophobic neutral OM are not always separated in literature,^18^ they were taken together as FA. The reactive metal content (M_react_) was based on a 0.43 M HNO_3_ extraction.^23^ Dissolved elements and pH were measured in a 1:10 solid-solution 10 mM Ca(NO_3_)_2_ extraction. The set of soils (Table S1.1) spans a wide range of pH (4.2–7.5), total solid and dissolved organic carbon (3.40–120 g kg^–1^ and 3.10–284 mg L^–1^), and M_react_ (Cd, Cu and Zn respectively 0.02–33.7, 1.31–318 and 0.97–24,021 mg kg^–1^).

### Model Framework

Our MSM featured the GTLM and NICA-Donnan submodels, with the addition of the Donnan model for clay particles.^24^ For metal-oxides, the more advanced CD-MUSIC model is expected to perform better in multisurface applications, especially regarding its ability to describe electrostatic interactions.^17^ However, a database of consistently derived CD-MUSIC parameters for our three cations is not yet available. We did not include uncertainty in the Donnan model for humic substances, since this would require refitting NICA proton parameters, which was outside the scope of our study. For clay, we used illite as reference clay with a surface charge of 0.25 equiv kg^–1^.^17^ Although this is a relatively high charge and illite may not be representative of clay minerals in all soils, we found that <3% of each metal cations was predicted to be bound to clay, corresponding to previous studies.^25^ We kept clay in our framework but did not evaluate the effect of uncertainty in clay surface charge. Precipitation mechanisms were not included, yet preliminary model calculations showed that including minerals containing Cd, Cu, Zn, Ca and PO_4_ had a limited influence on predicted concentrations (median changes −0.05%, −0.02% and −0.27% for Cd, Cu and Zn respectively), except for a contaminated soil close to a former Zn smelter with high M_react_ (2.7–53% decrease in dissolved metals).^18^

### Quantifying Model Parameter Uncertainty

The full descriptions of the NICA-Donnan model and GTLM are given elsewhere.^3,7^ To capture uncertainty related to the heterogeneity of isolated OM or synthesized metal-oxides, which is likely underestimated by fitting software when all data are fitted together (see Table S8.1), we quantified uncertainty in model parameters by calculating the average and sample standard deviation (SD) of the fitted individual metal binding data sets.^6^ For the GTLM (SI.6), we considered uncertainty in the binding constants for the strong and weak surface sites (log *K*_1_ and log *K*_2_), for which fitted values were taken from Dzombak and Morel^3^ for HFO. For the NICA-Donnan model (SI.5), we considered uncertainty in the density of carboxylic and phenolic functional groups (Qmax_1_ and Qmax_2_), the metal binding constants (log *K*_1_ and log *K*_2_) and nonideality parameters (*n*_1_ and *n*_2_) for both HA and FA. For Qmax we obtained fitted values for individual HA and FA samples from literature.^10^ For log *K*_*i*_ and *n*_*i*_ we refitted with the PEST-ORCHESTRA software combination^12^ the individual data sets previously fitted all-together,^1,26^ adopting the database selection by Tipping et al.^26^ We also included newly published data sets^11,12,27−32^ (SI.7).

### New Generic NICA-Donnan Parameters

With the enlarged database we also derived new generic NICA-Donnan parameters by fitting all data per element simultaneously, without data set weighting.^1^ For Zn, we had to take a different approach since fitting all data was not possible without restricting the number of parameters to fit (for FA), or because it led to unrealistic parameter combinations (for HA). Instead, we took the fits of one individual data set for HA^11^ and one for FA,^12^ both of which had much wider range of free Zn^2+^ concentrations than previously available,^1^ to obtain the new generic parameters (see SI.7 for details on this irregular treatment of Zn data). These individual data sets were fitted with a lower RMSE than the Milne et al.^1^ fits (0.08 vs 0.46 for FA; 0.10 vs 0.17 for HA).

Due to low data availability, we estimated Al, Fe and Mn parameters using linear free energy relations (LFERs). We evaluated two strategies for constructing LFERs, specifically using the Irving–Rossotti slope^26,33^ or to the first hydrolysis constants,^1^ and found that the amount of variance explained was similar for all log *K*_*i*_ and *n*_*i*_ except for FA-log *K*_1_ for which the hydrolysis constant (*K*_OH_) performed better (SI.4). We thus used *K*_OH_-based LFERs except for Fe–FA binding, for which we used published values.^34^ We deviated from Milne et al.^1^ who employed LFERs to estimate *n*_*i*_ and *n*_*i*_ log *K*_*i*_, and instead estimated log *K*_*i*_ and *n*_*i*_ individually, since we observed that linear log *K*_OH_–log *K*_*i*_ relationships accurately described fitted log *K*_*i*_ values (SI.4).

### Uncertainty Analysis

The uncertainty analysis was performed by running the MSM for each soil with a random sample from the distributions of model parameters described above and of soil-specific input values (Monte Carlo sampling, sample length *N* = 2000). For the model parameters, we used truncated normal distributions within two SDs around the mean to prevent unrealistic parameter combinations.^13^ For Zn in NICA-Donnan, the generic values were taken as the average, and the SDs were assumed at values comparable to Cu and Cd (Table S8.1). For Qmax_*i*_ and *n*_*i*_ only one SD was used to have values >0 or between 0 and 1, respectively. For HFO the log *K*_2_ had too few individual values for Cd and Cu (SI.6), hence these constants were sampled from a uniform distribution within 1 log-unit around the generic value.^3^ We imposed metal-specific correlation coefficients between NICA-Donnan parameters when sampling (SI.3).

We included uncertainty in soil-specific input values by running two scenarios: a local “best-case” scenario in which all relevant values were measured, and a global “simplified” scenario in which assumptions were made regarding the amount of reactive surfaces. In both scenarios, the amount of HFO was assumed to have a SD based on an analytical coefficient of variation (CV) of 5%. In the local scenario, unrestricted normal distributions were used for the SSA (determined value and average observed CV of 7.5%), and for the amount of HA and FA in the solid and dissolved fractions (measured value and assumed CV of 5%).

In the global scenario, truncated normal distributions were used within two SDs calculated based on the soils included in this study. For the SSA this was 614 ± 416 m^2^ g^–1^ (within one SD to prevent negative values), which is close to the commonly assumed value of 600 m^2^ g^–1^.^3^ For HA and FA we first sampled the percentage of reactive OM [humic substances (HS): HA + FA] relative to total SOC or DOC [solid HS (SHS) mean ± SD of 50 ± 23%; dissolved HS (DHS) 30 ± 12%; Table S1.1]. Next, fractionation was sampled on the basis that SHS consists predominantly of HA (SHA: 74 ± 10%), and DHS primarily of FA (DFA: 96 ± 4%, within one SD to not exceed 100%). The sampled percentages were used to calculate SHA, SFA, DHA and DFA based on measured SOC and DOC per soil. The average SOC reactivity is in line with other studies,^35^ yet the average DOC reactivity is lower than the 40–100% assumed in literature.^6^ The difference may be attributed to the diversity of soil samples in our study, which predominantly feature tropical soils that have been shown to have a lower DOC reactivity, such as an average of 21% by Van Eynde et al.^17^

### Model Implementation

The MSM was implemented in the ORCHESTRA software.^36^ Models were run with measured concentrations of Ca, PO_4_, Al^3+^, Fe^3+^ and Mn^2+^ (redox reactions were not included), and a fixed NO_3_ concentration (0.02 M). We did not include competing cation parameter uncertainty, because we found that predictions of Cu, Cd or Zn speciation were not sensitive to parameter uncertainties of the other two elements (not shown). Future studies can corroborate the limited influence of competing cations on MSM prediction uncertainty. We assumed an ambient pCO_2_ of 30 Pa for all soils. PO_4_ was measured as total-P, part of which likely exists in organic form. However, PO_4_ had a negligible effect on predicted concentrations; excluding PO_4_ led to +0.2%, +0.5% and +1.0% on average for Cd, Cu and Zn, respectively. The negligibility of this effect with the GTLM (i.e., excluding metal–PO_4_ ternary complexes) has also been observed with the CD-MUSIC model.^37^

The MSM calculated the distribution of elements over five species: bound to clay, HFO, SHA and SFA, or dissolved. Complexed dissolved species (DHA and DFA) were not analyzed separately. The predicted dissolved concentrations were compared with measured concentrations in 10 mM Ca(NO_3_)_2_. Over- or underprediction was quantified with the mean error (ME): average of log(modeled)–log(measured). Overall accuracy was quantified with root mean squared error (RMSE). Uncertainties in the predicted species were calculated as the interquartile range (IQR: width of the 25th–75th percentile range) of the percentage distributions of M_react_. Finally, we tested whether the IQR was significantly related to pH, SOM and M_react_ (SI.2).

### Sensitivity Analysis

In part because MSM predictions are based on equilibrium calculations, quantifying the sensitivity of MSM predictions to model parameters and input values is not straightforward. We used an *approximate* approach^38^ that consisted of analysis of variance decomposition of a first-order linear model per soil, per metal and per dependent variable (log-transformed concentrations of solid-phase species and total dissolved metal), in which model parameters and input values were the independent variables. Since the NICA-Donnan parameters were correlated in the uncertainty analysis, we resampled (*N* = 2000) all parameters without correlation coefficients to ensure stochastic independence. Sensitivity was quantified with two statistics: the top marginal variance (TMV), calculated as the total variance explained by an independent variable, and the bottom marginal variance (BMV), calculated as the variance that remains when excluding that variable from the full model.^38,39^ The TMV and BMV are complementary, however due to potential emergent correlations, we evaluated both statistics, where larger values indicated a higher sensitivity. The statistical analyses, the uncertainty analysis and the sensitivity analysis were performed in the R Statistical software.^40^

## Results and Discussion

### New Generic and Average NICA-Donnan Parameters

**Figure 2:**
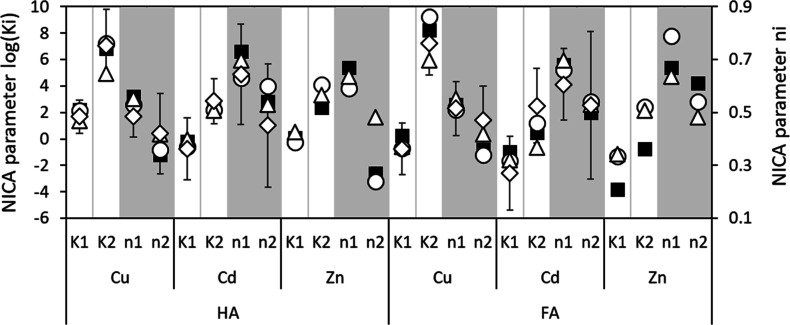
NICA log *K*_*i*_ (left) and *n*_*i*_ (right; shaded) values from Milne et al.^1^ (■) and obtained in this study as generic (◯), average (◇; with two SDs for log *K*_*i*_ and one for *n*_*i*_), or based on LFERs (△). For Zn the averages are the same as the generic values, see text.

The new generic parameters (Table 1) for HA were similar to those derived by Milne et al.:^1^ the differences in log *K*_*i*_ were <0.5 and in *n*_*i*_ < 0.1, except for the Zn-log *K*_2_ which increased by ∼1.5 log-units. For FA, the log *K*_1_ decreased by about 0.9 log-units for Cu and 0.7 log-units for Cd, whereas the log *K*_2_ increased by about the same amount (Figure 2). The new FA values thus made a stronger separation in binding strengths between carboxylic and phenolic groups, while heterogeneity (*n*_*i*_) remained similar. For Zn, both FA-log *K*_*i*_ were ∼3 log-units higher and corresponded with the LFER-derived values (Figure 2).

**Table 1:** New Generic and Average NICA-Donnan Parameters^a^

		log *K*_1_	*n*_1_	log *K*_2_	*n*_2_
Fulvic Acids					
generic	Al*	0.39	0.41	12.3	0.31
	Cd	–1.64	0.66	1.22	0.54
	Cu	–0.67	0.51	9.24	0.34
	Fe(III)**	2.70	0.36	8.30	0.23
	Mn*	–1.79	0.72	–1.88	0.55
	Zn	–1.29	0.79	2.41	0.54
average	Cd	–2.59	0.61	2.49	0.53
	Cu	–0.77	0.51	7.22	0.47
Humic Acids					
generic	Al*	2.74	0.41	7.64	0.31
	Cd	–0.61	0.63	2.23	0.60
	Cu	2.15	0.53	7.22	0.36
	Fe(III)*	4.27	0.26	10.7	0.20
	Mn*	–0.33	0.72	1.59	0.55
	Zn	–0.25	0.59	4.09	0.24
average	Cd	–0.75	0.64	2.86	0.45
	Cu	1.69	0.49	7.08	0.42

a Our new generic values were obtained by fitting all datasets together, or estimated with LFERs (*). The comparison with the previous parameters by Milne et al.^1^ is made in Figure 2. Average values were obtained by fitting individual datasets. The Fe(III) parameters for fulvic acids (**) were taken from Hiemstra and van Riemsdijk.^34^ For Zn the average values were the same as the generic ones (see text).

The average parameters for HA were comparable to the generic ones (Table 1). For FA the differences were larger, especially for the Cd-log *K*_1_ and Cd-log *K*_2_, which were respectively about 1 log-unit lower and higher compared to the generic values. The average Cu-log *K*_2_ was 2 log-units lower. For Cu it is known that there is substantial bi- or tridentate binding,^29,41−43^ and binding to functional groups containing nitrogen or sulfur.^44^ These higher-affinity binding mechanisms can only be characterized adequately if sorption data extend to low free concentrations and high pH.^1^ This may not have been sufficiently achieved for individual data sets, which could explain why average values differed from generic values.

Our LFER-based Mn parameters were almost identical to those by Milne et al.,^1^ because the differences between our linear log *K*_OH_–log *K*_*i*_ versus the exponentiated^1^ log *K*_OH_–log *K*_*i*_^*n*_*i*_^ were small within the calibration range of the LFERs in both studies (SI.4). For Fe^3+^ the log *K*_OH_ was outside of this range, and our log *K*_2_ was ∼7 log-units lower compared to Milne et al.^1^ For Al the parameters were previously fitted,^1^ but these log *K*_1_ values for HA and FA were 3–4 log-units lower than the LFER-values established here (Table 1), whereas the log *K*_2_ parameters were similar. We compared our parameters with those by Milne et al.^1^ by evaluating their ability to describe Al adsorption data for isolated HA^26,45^ and FA.^26^ Our parameters led to slightly better predictions for HA and slightly worse for FA, yet the accuracy was generally low (*R*^2^ 0.53–0.64; RMSE 0.33–0.56), in agreement with previous studies.^1,46,47^ This inaccuracy may in part be related to the use of the Donnan model, which describes electrostatic interactions with insufficient accuracy, especially for trivalent cations.^47^ Alternative approaches such as employing a two-parameter Donnan model^47^ or introducing cation-selectivity^46^ could improve descriptions of Al adsorption. We present our LFER-based estimates of Al-log *K*_*i*_ to meet the recognized need to increase Al competition effects (see SI.4 for more details).^47^ Our Al parameters led to a higher model accuracy for Cd, Cu and Zn (Table 2: local scenario, present study), since the parameters of Milne et al.^1^ would result in higher RMSEs (0.02–0.08 log-units, not shown).

**Table 2:** Accuracy of Multisurface Model Predictions of Dissolved Concentrations Compared with Measured Concentrations in 10 mM Ca(NO_3_)_2_, Quantified by the Root Mean Squared Error (RMSE) and Mean Error (ME)^a^

scenario		cadmium		copper		zinc	
		RMSE	ME	RMSE	ME	RMSE	ME
Generic Parameters from Milne et al.^1^ and from the Present Study (Table 1)							
local	Milne et al.^1^	0.56	0.29	0.40	0.09	1.15	0.84
	present study	0.62	0.32	0.40	0.02	1.12	0.60
global	Milne et al.^1^	0.49	0.21	0.45	0.06	1.08	0.82
	present study	0.50	0.26	0.44	0.00	1.05	0.60
Average Parameters from Individually Fitted Data Sets (Table 1)							
local	single run	0.68	–0.11	0.49	–0.20	1.12	0.63
	mean (*N* = 2000)	0.56	0.09	0.46	–0.13	1.08	0.69
global	single run	0.55	–0.18	0.51	–0.22	1.06	0.63
	mean (*N* = 2000)	0.49	0.21	0.44	0.01	1.08	0.79

a The scenario refers to whether organic matter fractionation (into HA and FA) and metal-oxide SSA were measured (local) or assumed (global). For the average parameters the accuracy is given when the generic values were replaced by average values (single run), and when the model was run 2000 times with the sampled parameters and input values.

### Uncertainty in Model Parameters

The log *K*_*i*_ values for HA and FA had a standard deviation of 0.63–1.41 log-units (Figure 2), while the SD for *n*_*i*_ ranged from 0.08 to 0.15 for Cu and from 0.14 to 0.28 for Cd. The functional group densities were highly uncertain, especially Qmax_2_ with CVs of 59% (FA) and 61% (HA), which for carboxylic Qmax_1_ groups were 24% and 30% (Table S5.1). In addition to uncertainty in all NICA-Donnan parameters caused by natural variation in soil-specific HA and FA metal-binding, the relatively large uncertainty for Qmax_2_ is likely due to analytical challenges of carrying out acid–base titrations above pH 10.^41^

Ideally, proton parameters (including Qmax) and metal parameters would be determined for the same humic substance sample. Such data is scarce, and similar to Milne et al.^1^ we used fixed generic proton parameters when fitting metal parameters. It has been shown that metal parameters are mostly independent from Qmax, except at environmentally unrealistic high metal concentrations where saturation could occur,^1^ whereas the affinities of metals (log *K*_M_) and protons (log *K*_H_) are closely related.^26,33^ Consequently, log *K*_M_ uncertainties could be overestimated, since fitting observed metal adsorption curves at an assumed generic log *K*_H_ that is higher than the actual (unknown) value of the humic substance would overestimate log *K*_M_, and vice versa.

The uncertainty in the affinity to metal-oxides was relatively low, with log *K*_M_ SDs ranging from 0.23 to 0.34 log-units (SI.6). This seems particularly low considering the variability in the SSA of oxides synthesized in the laboratory.^48^ This variability was not included when fitting individual data sets, instead a generic SSA of 600 m^2^ g^–1^ was used as previously recommended.^3^ Deviation between the real and assumed SSA of individual metal-oxide samples would lead to under or overprediction of log *K*_M_, indicating that using sample-specific SSA values would likely reduce the uncertainty of these constants. For both HFO and OM, most of the average parameters were well within one standard deviation of the generic values (Figure 2, Table S8.1).

### Accuracy of Model Predictions

**Figure 3:**
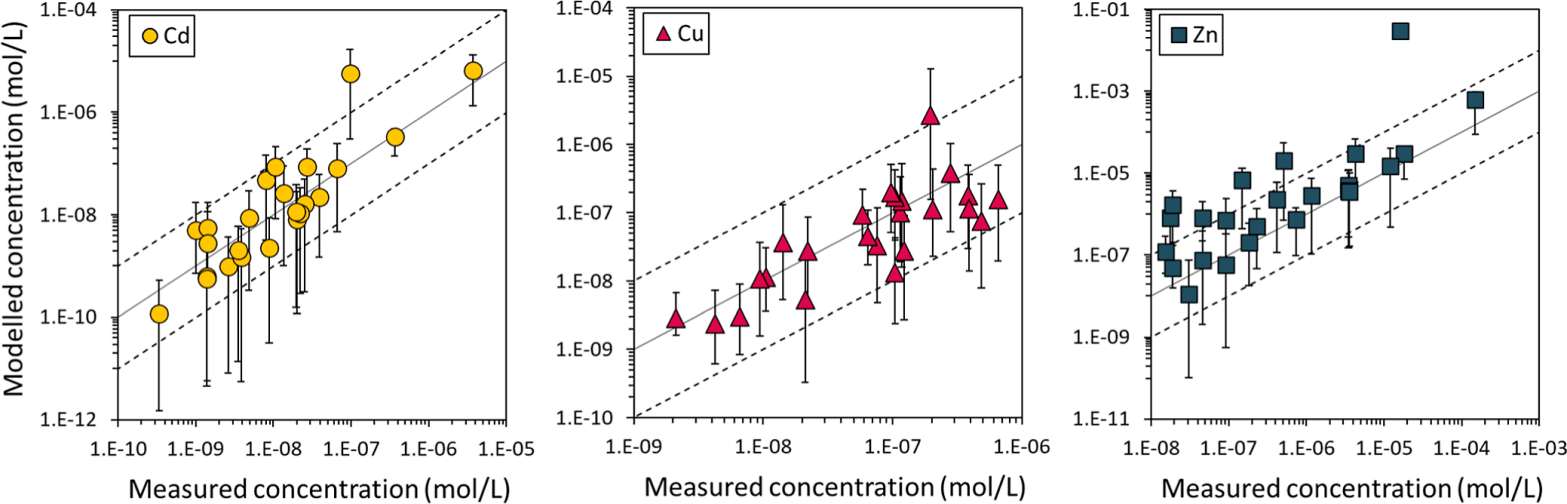
Model assessment of the average (*N* = 2000) predicted dissolved concentrations versus measured concentrations in 10 mM Ca(NO_3_)_2_ in the local uncertainty analysis for cadmium (left; ◯), copper (middle; △) and zinc (right; □). The lower and upper error bars are the 2.5th and 97.5th percentiles, respectively, thus showing the 95% prediction intervals. Dashed lines represent 1 log-unit around the 1:1 line.

The accuracy of predicted dissolved Cd and Cu concentrations was similar when using the new generic NICA-Donnan parameters compared to using those by Milne et al.^1^ (Table 2). For Zn the accuracy improved substantially with the new parameters, with overprediction (ME) decreasing by 0.24 log-units (Table 2), which can be attributed to the higher log *K*_*i*_ compared to Milne et al.,^1^ providing further evidence that our new generic parameters can improve MSM predictions (Table 1). Using average parameters had a negligible influence on model accuracy, except for a noticeably larger absolute ME for Cu, which became underpredicted (Table 2). For all three elements, one soil close to a former Zn smelter with extremely large M_react_ values was a clear outlier irrespective of which parameters were used (Figure 3).

The 95% prediction interval (PI) in the local scenario showed that relative to the average, the PI mainly extended to lower dissolved concentrations, especially for Cd and Zn (Figure 3), as evidenced by the larger absolute difference between the average-2.5th percentile compared to the average-97.5th percentile: for Cd these values were 1.45 and 0.50 and for Zn 1.10 and 0.42 log-units, respectively (for Cu they were more similar: 0.76 and 0.50). This tendency to predict lower dissolved Cd and Zn corresponded with the observations that these concentrations on average were overpredicted (Table 2), suggesting that model parameter uncertainty explains part of the prediction inaccuracy.

It is highly relevant to note that across all analyses (previous and new generic parameters, and average parameters) there was little difference in model accuracy between the local scenario and the global scenario (Table 2). In other words, Cd, Cu and Zn solid-solution partitioning can be accurately modeled with basic soil properties (e.g., total SOC and DOC) and the assumptions for reactive surfaces presented in this study (regarding reactive OM and metal-oxide SSA).

Whereas MSM-based solid-solution partitioning can be validated by comparing predicted and measured dissolved concentrations, the limited empirical results in literature (e.g., based on spectroscopy) currently do not provide adequate validation of speciation predictions. For Cu and Cd several studies demonstrated OM to be an important surface.^49,50^ However, for Zn clay and metal-oxides have been found to dominate speciation,^50,51^ yet results seem to differ per soil type and are likely related to the natural or anthropogenic source of this metal. The lack of adequate validation of predicted speciation must be kept in mind when interpreting the uncertainty and sensitivity analyses, and presents a knowledge gap to be addressed in future studies.

### Uncertainty of Model Predictions

**Figure 4:**
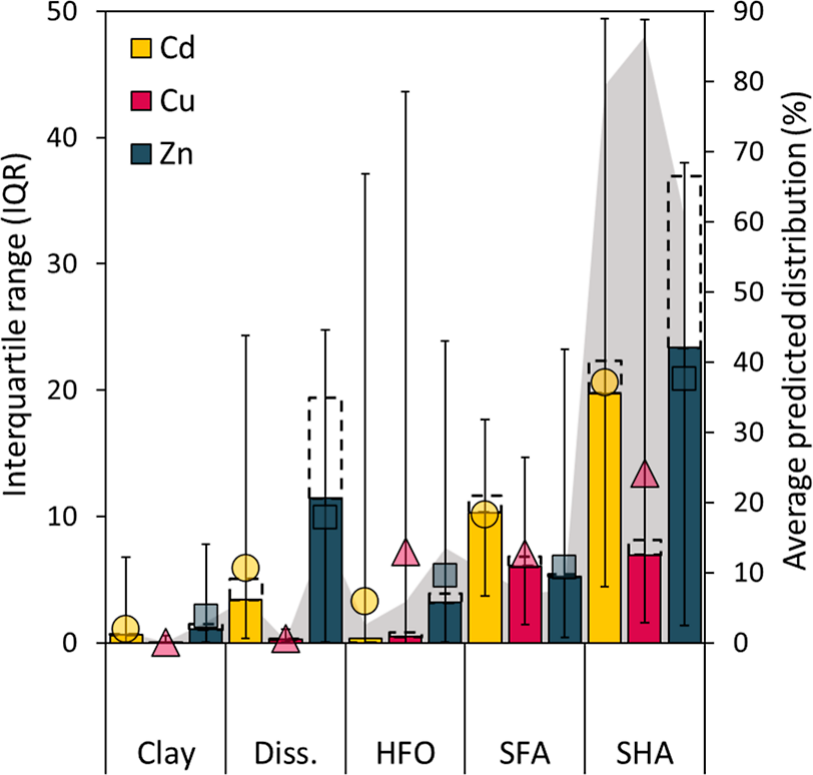
Uncertainty in predicted speciation (percentage of M_react_) quantified with the interquartile range (IQR, left axis). The figure gives the median (filled bars), average (symbols), minimum and maximum (error bars) IQR of all soils in the local scenario. The shaded area in the background gives the average speciation of all soils (percentage of M_react_, right axis) to facilitate interpretation. For comparison, the dashed bars represent the median IQR in the global scenario.

Prediction uncertainties varied strongly between soils (Figures 4, S2.1–S2.6). Unless mentioned otherwise, the results presented are based on the local scenario. As expected, all three elements were predicted to be mostly bound to solid organic matter, with on average 61–86% SHA-bound and 7.2–10% SFA-bound (Figure 4). For SFA the uncertainty was similar for all three elements, whereas for SHA the uncertainty was about three times larger for Cd and Zn (median SHA-IQR 20 and 23%, respectively) compared to Cu (7%). With high certainty (median Clay-IQR <1.2%), clay was predicted to adsorb very little of all three elements (<3% on average). Dissolved Cd and Zn concentrations were higher (on average 6.3% and 16% of M_react_) than dissolved Cu (<0.5%), with soil-dependent uncertainties (dissolved-IRQ) decreasing significantly with pH, M_react_ and SOM (Table S2.1). For neutral to alkaline soils HFO becomes an important surface,^2^ corresponding to our observation that the HFO-IQR increased significantly with pH and decreased with SOM (Table S2.1).

For Cu, MSM uncertainty was mainly about the uncertainty in solid-phase speciation. The 95% prediction intervals (PIs) clearly showed SHA to be the major binding surface, but for high-pH and low-SOM soils it often could not be predicted with confidence whether the remaining part was HFO- or SFA-bound, as the PIs overlapped (Figure S2.3). Such overlap was also observed for Cd and Zn. Additionally, for both elements the predicted dissolved concentrations varied strongly between soils, as did their uncertainties (Figure 4), which decreased significantly with pH, SOM and M_react_ (Table S2.1). The effect of model parameter uncertainty on Cd and Zn speciation uncertainty thus depends on multiple soil properties.

For most soils, Cd was predominantly bound to SHA (Figure S2.1). The amount bound to SFA was rather constant over all soils (10 ± 4%), with constant uncertainty (SFA-IQR 10 ± 3%). However, the PI of SFA overlapped with both HFO (for high-pH soils around 7.5) and dissolved species (for low-pH soils in the range of 4.2–4.9, with Cd_react_ of ∼0.05 mg kg^–1^). This difficulty of predicting with confidence whether Cd was SFA-bound or dissolved was not found when soils, although equally low in pH, contained more OM and Cd_react_ (0.5–2 mg kg^–1^).

For Zn, HFO became a relevant surface at lower pH values than was observed for Cd. For several low-OM soils with pH 6.1–6.7, we could not predict with 95% certainty whether the majority of Zn was HFO- or SHA-bound. Similar to Cd, for soils with low Zn_react_ and pH we found a relatively uncertain dissolved fraction (dissolved-IQR 3.2–25%). Furthermore, Zn was the only element for which the 95% PIs of SHA and SFA overlapped for a diversity of soils. Hence, the uncertainty in MSM parameters implies that although most Zn is predicted to be OM-bound, especially at pH < 6.5, we cannot predict with 95% certainty whether most is bound to solid humic or fulvic acid.

Naturally, in the global scenario the uncertainties generally became larger for all metal-species, although the average model accuracy did not necessarily worsen (Table 2). Nevertheless, this increase in uncertainty was rather small, with IQRs for most metal-species increasing between <0.01 and 2.6 percentage-points relative to the local scenario (Figure 4). The implications of the assumptions about the amount of reactive surfaces were larger for Zn, for which HFO was an important surface, than for Cd and Cu, which were dominated by OM binding. Consequently, the Zn Dissolved-IQR and SHA-IQR increased strongly (7.9 and 13.6 percentage-points, respectively). Compared to the IQRs, the 95% PIs showed larger increases (up to 20 percentage-points), indicating that the global scenario led to more extreme (high and low) predictions (Figure S2.7).

### Sensitivity of Model Predictions

**Figure 5:**
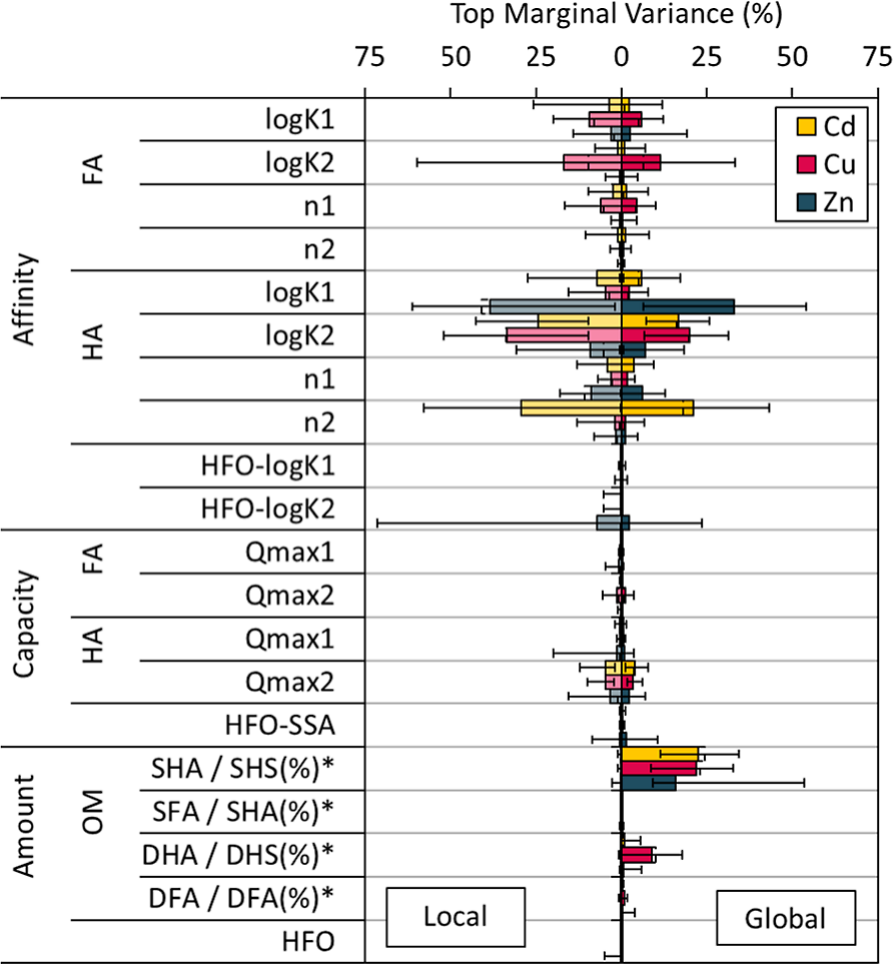
Sensitivity of predicted dissolved concentrations as quantified with the top marginal variance (TMV). For each parameter the average (filled bar), minimum and maximum (error bars) of all soils are shown. The amount of OM (*) is measured in the local scenario (left, in kg kg^–1^ or kg L^–1^) and assumed in the global scenario (right, in percentage of total SOC or DOC, see text).

We first present the local scenario sensitivity analysis for the dissolved metal concentrations (Figure 5). The other species were found to have a similar sensitivity profile (not shown), indicating that this analysis is applicable to the overall MSM approach. We focus on the TMV, since these values were similar to the BMV, indicating little emergent correlations between parameters and input values (Figure S2.8).

Uncertainties in metal-oxide related parameters had only a minor impact on prediction uncertainties (Figure 5), due to the large number of binding sites on HFO relative to the adsorbed amount of metals. Only for Zn in high-pH (>6.5) and high Zn_react_ soils (>approximately 100 mg kg^–1^) did uncertainty in HFO affinity become relevant, particularly the strong site log *K*_2_. We had three such soils, for which Zn-log *K*_2_ explained 31–72% of the variance (TMV) in the dissolved concentration. Another soil (pH 7.1) had such high Zn_react_ (24,021 mg kg^–1^) that 58% and ∼100% of the weak and strong HFO sites were occupied, respectively. Only for this soil did capacity parameters play a role: the SSA–TMV was 8% and analytical uncertainty in the measured amount of HFO explained 5%. Hence, for acidic to neutral soils with background Zn levels, uncertainties related to HFO will not influence model predictions. Even if such soils have substantial HFO-binding, the uncertainty therein will instead be explained by uncertainty in OM parameters.

Model predictions were most sensitive to uncertainty in NICA parameters for all three metals (Figure 5). Despite similar uncertainties in FA and HA parameters (Figure 2), the sensitivity was especially high for HA affinity parameters, due to SHA generally being the major binding surface. Because the MSM is additive, uncertainty in HA parameters tended to dominate the uncertainties in all modeled species. Between the HA affinity parameters, dissolved Zn was most sensitive to the carboxylic log *K*_1_, whereas Cd and Cu were more sensitive to the phenolic log *K*_2_ (Figure 5), potentially because phenolic groups become more important at low concentrations (average pCd, pCu and pZn were 8.0, 7.2 and 6.3, respectively). Because 79% of dissolved Cu was DFA-bound, compared to 15% and 2% for Cd and Zn, respectively, the predicted total dissolved Cu concentrations were also sensitive to the FA affinity parameters (Figure 5).

Model predictions were not sensitive to the NICA capacity parameters Qmax_*i*_ (Figure 5). Although the range in Qmax_*i*_ was large (4.9–9.6 and 3.2–8.0 mol kg^–1^ for FA and HA, respectively, Table S5.1), the same capacity was assigned to SHS and DHS, thus leveling out the effect of increasing Qmax_*i*_ in the solid phase (more adsorbed) and the dissolved phase (more dissolved). Since log *K*_M_ parameters are relatively independent of Qmax,^1^ by sampling capacity and affinity parameters independently we showed that uncertainty in model predictions is determined by uncertainties in log *K*_M_ and not in the amount of functional groups. The prediction uncertainties could be overestimated, because log *K*_M_ was sampled at constant log *K*_H_. Nevertheless, assuming log *K*_M_ and log *K*_H_ are correlated, the sensitivities described here would remain similar, as statistically the variation in predicted concentrations could be explained by both affinities.^26,33^

In the global scenario, the sampled affinity parameter uncertainties were identical to those in the local scenario, and model predictions remained sensitive to them (Figure 5). In addition, the assumption on the amount of solid OM to be reactive (SHS %) became influential, with TMV between 6 and 38% across soils. Whether this reactive OM was interpreted as HA or FA (SHA %) had little to no effect (TMV <0.5%). Only for Cu did it matter how much of the dissolved OM was assumed reactive (DHS %), which explained on average 9% of the variance for all soils. Since 96 ± 4% of reactive DOC was DFA as measured for all soils, this small variation did not explain variance in the predicted amount of dissolved Cu (TMV <1%).

### Implications for Geochemical Modeling

Our new generic NICA parameters substantially improved the accuracy of MSM predictions, particularly for Zn. Future investments in MSM improvement should focus on HA affinity parameters, since these had the largest influence on prediction uncertainty. Specifically, a quantitative understanding is needed of the element-specific relationships between metal and proton affinity parameters. Currently, the 95% prediction interval of dissolved Cd, Cu and Zn is about 1 log-unit, with predictions becoming more uncertain at low pH, SOM and M_react_. Depending on the environmental purpose of MSM employment, this is important to consider. At some point, the use of *generic* parameters will limit how much MSM predictions can be improved further, given the range of systems they aim to describe. Then, with OM being such an important surface, reducing model uncertainty requires knowledge of the soil-specific composition and binding properties of organic matter.

We established simplifying assumptions about soil surface reactivity,^18^ based on a uniquely diverse set of soils from contrasting climatic and geographic regions: 50% of SOC reactive, 74% of which SHA; 30% of DOC reactive, 96% of which DFA; oxide SSA 614 m^2^ g^–1^. These assumptions had a negligible impact on model accuracy and uncertainty, thus enhancing the wide-scale applicability of MSMs with the NICA-Donnan model and GTLM, since soil OM is often measured and available from soil maps,^2^ and the SSA is often unknown whereas Fe and Al (for calculating the amount of HFO) are often measured.^9^ By comprehensively presenting model performance regarding accuracy, uncertainty and sensitivity, our study makes a mechanistic approach to understanding the solid-solution partitioning of heavy metals in soils an accessible alternative to empirical models.^2,52^ This is essential for enabling the use of geochemical models in addressing societal challenges, such as the development of environmental protection criteria.^4,53^

## Supporting Information Available

PDF with supporting results and justifications of all choices made for the analyses carried out in this study (PDF)
