# Supplementary material for: Quantifying the Accuracy, Uncertainty, and Sensitivity of Soil Geochemical Multisurface Models
Source: Environ Sci Technol. 2025 Mar 5;59(10):5172–81. doi: 10.1021/acs.est.4c04812 (PMC11924228; doi:10.1021/acs.est.4c04812)
Supplement: Supplementary file 1 — es4c04812_si_001.pdf [file es4c04812_si_001.pdf]

<u>Supporting Information</u>

Quantifying the accuracy, uncertainty and sensitivity of soil geochemical multi-surface models

Wietse Wiersma [1,2], Elise van Eynde [3], Rob N.J. Comans [1], Jan E. Groenenberg [1]*

[1] Soil Chemistry Group, Wageningen University & Research, 6708 PB Wageningen, the Netherlands

[2] Soil Biology Group, Wageningen University & Research, 6708 PB Wageningen, the Netherlands

[3] European Commission, Joint Research Centre (JRC), 21027 Ispra, Italy

Summary: 43 pages, 15 figures, 14 tables

Contents:

SI.1 Soil properties

- Table with measured soil properties (page S3-S4).
- Explanation of calculation of amount of HFO (page S5).

SI.2 Additional results

- Six figures with predicted speciation per element and per soil, for local and global scenarios (page S6-S8).
- Figure with uncertainty quantified with the 95% prediction interval (page S9).
- Figure with sensitivity profile (including both TMV and BMV values) (page S10).
- The statistical significance of soil properties explaining uncertainty (IQR) (page S11).

SI.3 Correlation matrices NICA parameters

- Explanation of how correlation matrices were obtained, and how they were implemented during the Monte Carlo sampling approach (page S12).
- Table with correlation matrix (page S13).
- A discussion about the effect of including or excluding the correlation matrix between NICA parameters on MSM uncertainty and sensitivity (page S14).
- Figure with uncertainty (IQR and 95% prediction interval) for the local scenario without correlation between NICA parameters (page S15).
- Figure with sensitivity profile (including both TMV and BMV values) for the local scenario without correlation between NICA parameters (page S16).

SI.4 Linear free energy relationships (LFERs)

- Comparison between the use of Irving-Rossotti slope and first hydrolysis constant for the construction of linear free energy relationships (page S17-S18).
- Two figures demonstrating available data to construct LFERs and the points considered outliers; comparison between our approach and that of Milne et al. (2) (page S19).
- Table with estimates of NICA constants based on LFER (page S20).
- Comparison between our LFER-based Al parameters with those fitted by Milne et al. (2) in their ability to describe adsorption to isolated humic and fulvic acids. Including two figures relating differences to Al concentration, pH and ionic strength (page S21-S23)

SI.5 Uncertainty in organic matter functional group density (Qmax)

- Explanation of how we derived the uncertainty in Qmax parameter (page S24).
- Table and figure showing available data and the generic and average data that were used in our study, and how they were obtained (page S25).

SI.6 Uncertainty in metal-oxide binding parameters

- Explanation of how we derived the uncertainty in HFO/GTLM parameters (page S26).
- Table with generic and average HFO/GTLM parameters used in our study (page S27).

SI.7 Annotated summary of individual NICA-Donnan database fits

- Explanation of how we derived new generic NICA-Donnan parameters and how we established the average and standard deviation. Explanation of the information provided in the following tables that present the fitting results of each individual dataset (page S28).
- Table presenting the source of each dataset used in this study (page S29).
- Six tables showing all NICA-Donnan fitting results per element and per humic substance (fulvic acid and humic acid) (page S30-S39).

SI.8 Summary sampling approach local and global uncertainty analysis

- Table with an overview of all model parameters (generic and average) and soil-specific input values (measured or estimated), including the distributions from which they were sampled during the local and global scenarios (page S40-S41).

SI.1 Soil properties

Table S1.1. Soil properties. Solid and dissolved organic carbon (SOC and DOC) were fractionated into humic and fulvic acids (HA and FA). Metal oxides were calculated (see below) based on measured concentrations in ammonium oxalate (AO) and dithionite (D) extractions. For the reactive surface area (RSA) and specific surface area (SSA) also the standard deviation is given (±). See main text for details.

Soil	pH	Clay	SOC			DOC			Metal oxides				RSA	±	SSA	±	HNO_3 extraction		
			Tot.	HA	FA	Tot.	HA	FA	AO-Fe	AO-Al	D-Fe	D-Al					Cd	Cu	Zn
		%	$g\ kg^{-1}$			$mg\ L^{-1}$			$mmol\ kg^{-1}$				$m^2\ g^{-1}$				$mg\ kg^{-1}$		
**CH-L2**	7.5	33	31	4.0	2.2	12	0.24	4.1	51	61	1003	248	5.6	0.6	581	56	1.31	25.6	234
**CH-H2**	7.1	9	122	5.6	2.1	6.4	0.06	2.6	296	120	722	147	8.5	1.0	243	27	30.2	318	24021
**CH-L4**	7.5	44	25	3.1	2.5	8.4	0.08	2.5	40	76	1101	281	16	0.8	1363	49	2.09	14.5	77.9
**CH-H4**	5.7	15	39	5.9	2.5	13	0.13	5.8	65	56	423	83	3.5	0.2	341	22	33.7	41.5	1039
**CH-L5**	4.6	40	13	2.7	1.6	3.1	0.16	1.8	52	86	1929	422	23	0.7	1589	37	1.10	17.6	43.5
**CH-H5**	6.4	29	23	4.4	1.8	3.1	0.12	1.5	100	117	1429	337	13	0.1	695	3.8	3.57	79.3	425
**EC-AM1**	7.1	15	19	12	4.2	60	0.98	16	111	150	197	121	5.7	0.3	263	11	0.61	16.2	13.1
**EC-AM2**	5.0	18	20	9.3	2.8	12	0.21	2.5	95	331	345	284	26	2.5	693	60	0.13	8.04	11.4
**EC-COA1**	5.2	29	17	5.9	2.2	26	0.33	4.7	194	51	566	95	13	2.8	565	114	0.66	14.4	16.7
**EC-COA2**	6.7	39	11	5.5	2.1	245	0.13	9.2	170	89	446	102	5.7	0.2	261	10	1.05	24.6	33.1
**COL**	6.2	9	4.4	2.1	0.7	3.3	0.16	0.7	30	15	243	51	0.8	2.5	164	592	0.67	1.31	5.94
**BI-M3**	4.3	7	50	23	2.4	18	0.33	4.3	156	314	459	329	16	0.1	393	1.2	0.04	3.12	1.32
**BI-M5**	4.4	10	28	7.7	3.0	15	0.21	4.1	71	168	492	245	17	0.6	797	24	0.03	6.62	1.98
**BI-M14**	4.2	17	22	8.0	1.6	12	0.13	3.6	58	148	647	253	28	0.1	1387	3.2	0.03	3.19	0.97
**BI-M16**	4.2	4	14	4.1	1.4	6.6	0.22	2.0	37	63	328	140	11	0.2	1154	20	0.02	3.77	1.39
**BI-R1**	4.9	8	32	14	1.2	6.8	0.08	1.4	66	136	730	172	10	0.2	595	9.2	0.08	5.16	1.04
**RW-R39**	6.0	8	40	9.4	0.8	34	0.03	3.9	31	43	616	153	7.0	2.2	998	286	0.05	3.88	6.58
**NL-T031**	4.4	41	61	32	9.5	284	0.35	71	306	145	338	128	8.1	0.6	216	15	0.49	15.8	23.9
**NL-T054**	4.5	8	95	65	11	275	0.44	83	232	96	220	97	5.0	1.5	181	51	0.62	18.5	83.5
**NL-T072**	4.8	1	36	23	7.5	223	0.36	65	144	61	190	73	6.9	0.1	390	4.1	0.49	18.1	23.9
**NL-T074**	5.6	1	33	9.0	7.2	39	0.37	17	8.0	47	8.6	45	4.0	0.2	829	39	1.95	2.74	93.5
**NL-T155**	4.4	5	64	39	11	184	0.19	48	448	67	969	100	16	1.6	355	35	0.49	7.17	21.0
**IE-G**	5.5	22	4.7	1.9	1.2	43	0.24	11	121	49	229	65	3.9	0.2	273	13	0.75	9.69	17.5
**IE-B**	6.1	20	3.4	1.7	0.9	25	0.21	6.6	102	49	328	87	5.2	0.6	399	44	1.23	17.3	11.5

Continued from previous page.

Soil	pH	Clay	SOC			DOC			Metal oxides				RSA	±	SSA	±	HNO_3 extraction		
			Tot.	HA	FA	Tot.	HA	FA	AO-Fe	AO-Al	D-Fe	D-Al					Cd	Cu	Zn
		%	g kg^{-1}			mg L^{-1}			mmol kg^{-1}				m^2 g^{-1}				mg kg^{-1}		
**Average**	5.5	18	34	12	3.5	65	0.24	16	124	106	582	169	11	0.8	614	64	3.39	28.2	1092
**Median**	5.3	15	26	6.8	2.2	16	0.21	4.2	98	81	453	134	8.3	0.6	482	26	0.64	14.5	19.3
**Min.**	4.2	1.0	3.4	1.7	0.7	3.1	0.03	0.7	8.0	15	9.0	45	0.8	0.1	164	1.2	0.02	1.31	0.97
**Max.**	7.5	44	122	65	11	284	0.98	83	448	331	1929	422	28	2.8	1587	592	33.7	318	24021

Calculation of amount of oxides (HFO)

We measured Fe and Al in ammonium oxalate (AO) and dithionite (D) extractions (Table S1.1). The concentrations in AO were used to calculate amorphous Fe and Al oxides, with molar masses of 95 and 84 g mol^{-1}, respectively. The difference between D and AO concentrations was used to calculate crystalline Fe and Al oxides, with molar masses of 89 and 78 g mol^{-1}, respectively. The molar amounts of Al and Fe oxides were added together and interpreted as hydrous ferric oxide (HFO), and $1/6^{th}$ of the crystalline was added to the amorphous amounts, as explained in Van Eynde et al. (3).

SI.2 Additional results

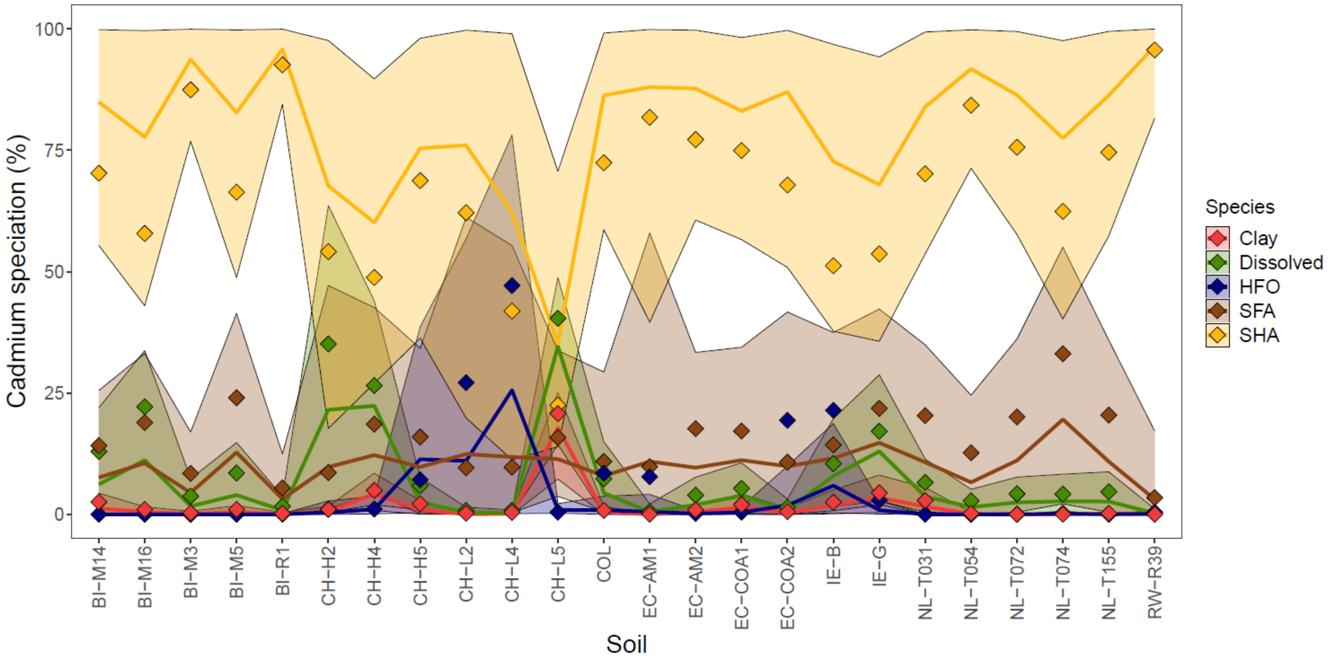

Figure S2.1. Predicted cadmium speciation (percentage of HNO_3 total) in the local uncertainty analysis. The thick lines are the averages of all model runs (N=2000) and the ribbon around it the 95% prediction interval. The diamonds (◇) are the speciation results with the new generic parameters in a single run.

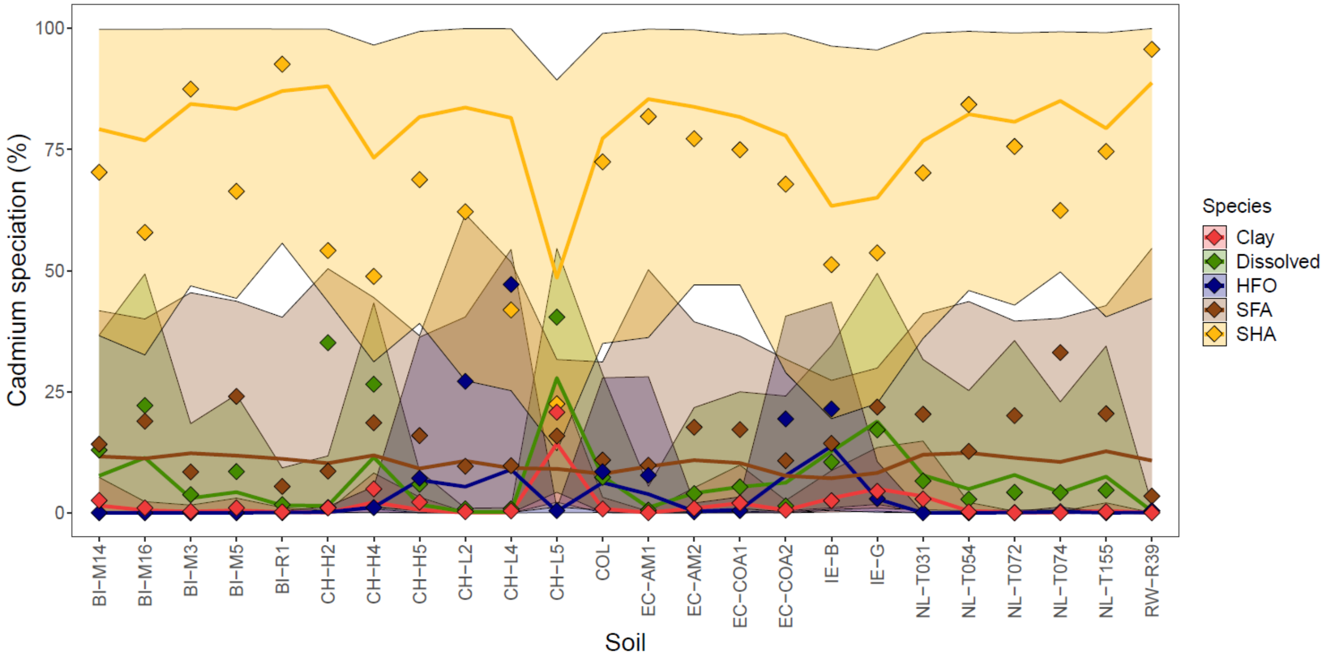

Figure S2.2. Predicted cadmium speciation (percentage of HNO_3 total) in the global uncertainty analysis. The thick lines are the averages of all model runs (N=2000) and the ribbon around it the 95% prediction interval. The diamonds (◇) are the speciation results with the new generic parameters in a single run.

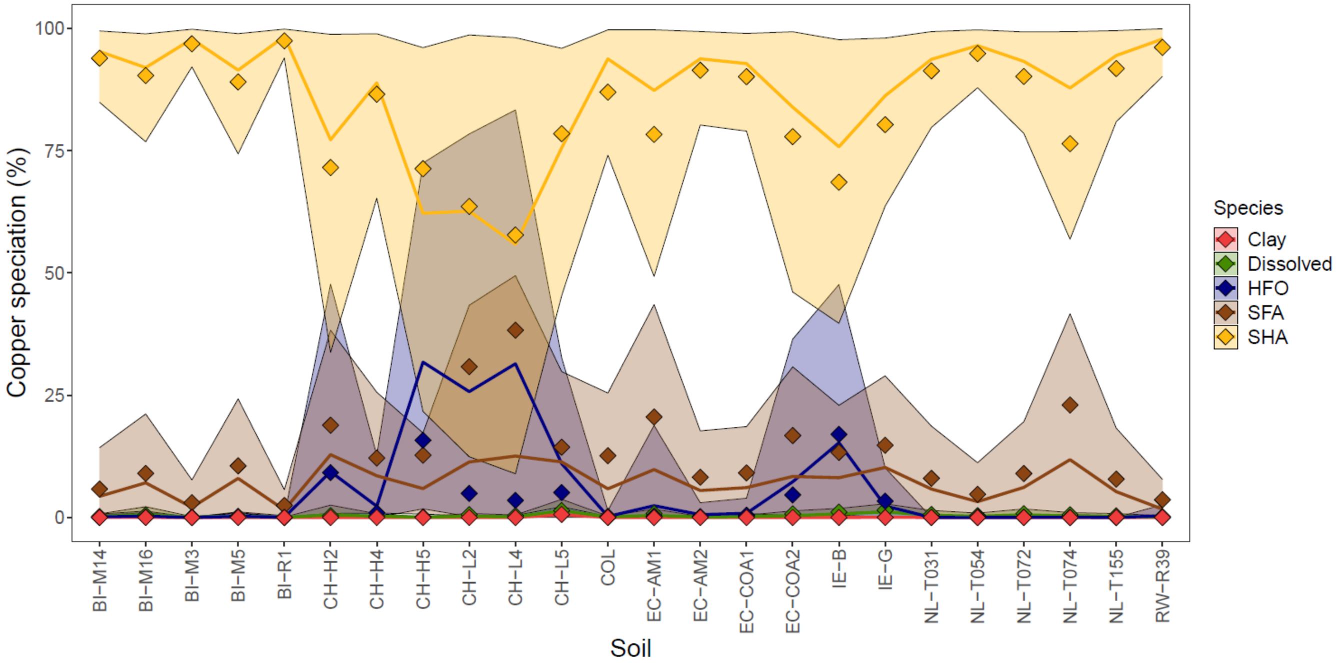

Figure S2.3. Predicted copper speciation (percentage of HNO_3 total) in the local uncertainty analysis. The thick lines are the averages of all model runs (N=2000) and the ribbon around it the 95% prediction interval. The diamonds (◇) are the speciation results with the new generic parameters in a single run.

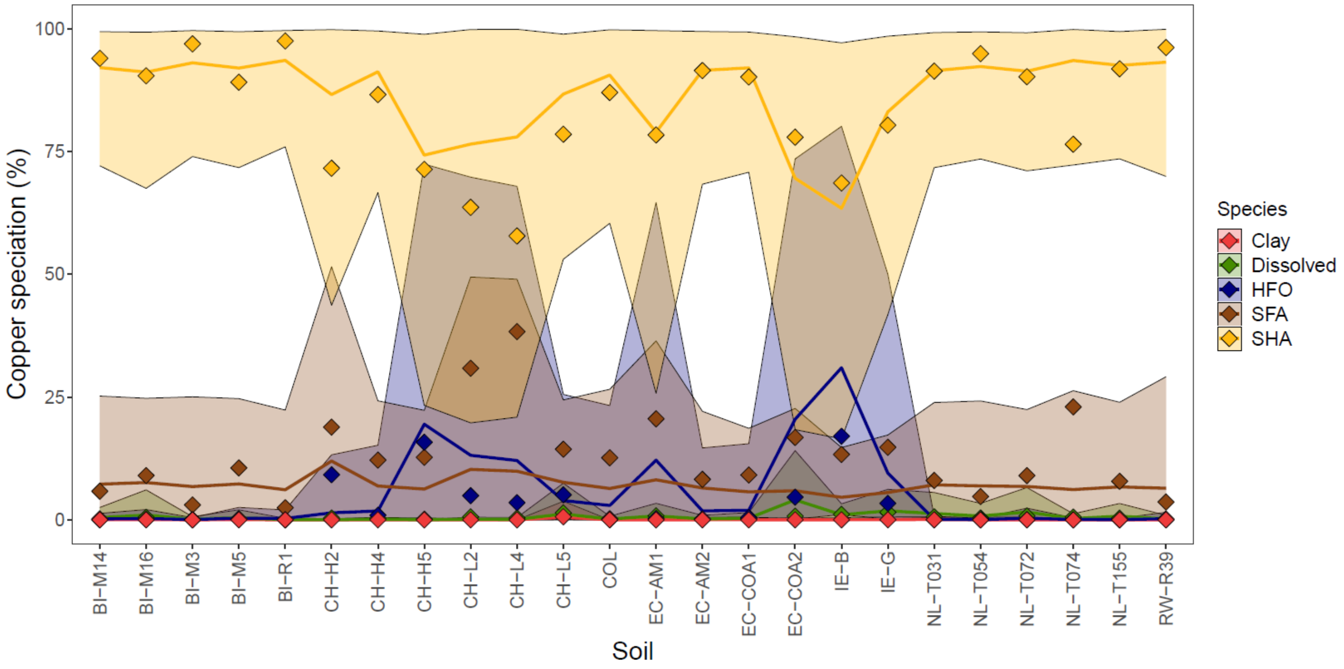

Figure S2.4. Predicted copper speciation (percentage of HNO_3 total) in the global uncertainty analysis. The thick lines are the averages of all model runs (N=2000) and the ribbon around it the 95% prediction interval. The diamonds (◇) are the speciation results with the new generic parameters in a single run.

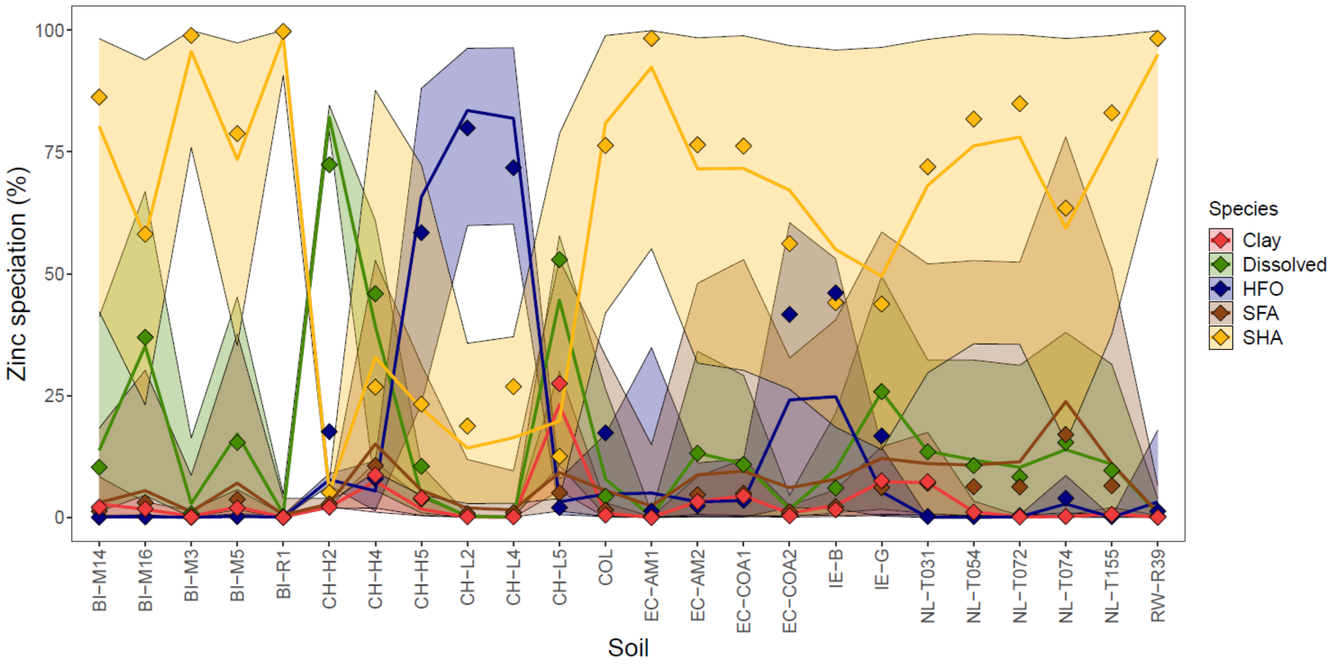

Figure S2.5. Predicted zinc speciation (percentage of HNO_3 total) in the local uncertainty analysis. The thick lines are the averages of all model runs (N=2000) and the ribbon around it the 95% prediction interval. The diamonds (◇) are the speciation results with the new generic parameters in a single run.

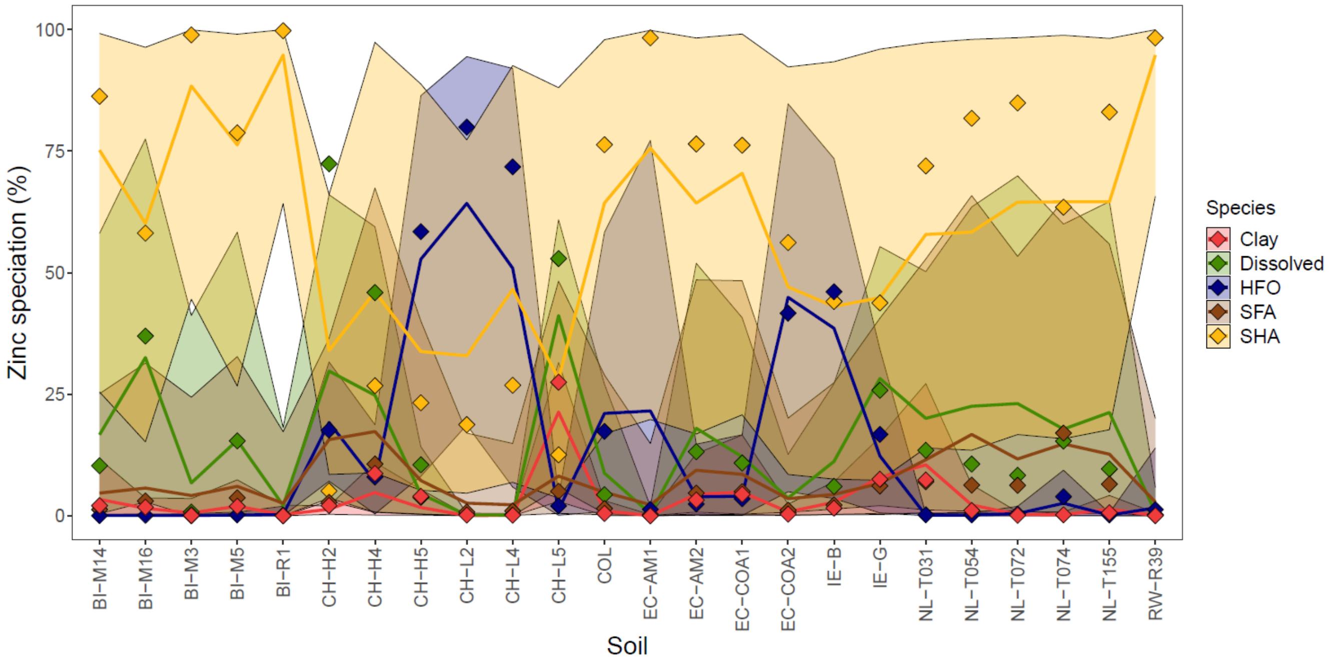

Figure S2.6. Predicted zinc speciation (percentage of HNO_3 total) in the global uncertainty analysis. The thick lines are the averages of all model runs (N=2000) and the ribbon around it the 95% prediction interval. The diamonds (◇) are the speciation results with the new generic parameters in a single run.

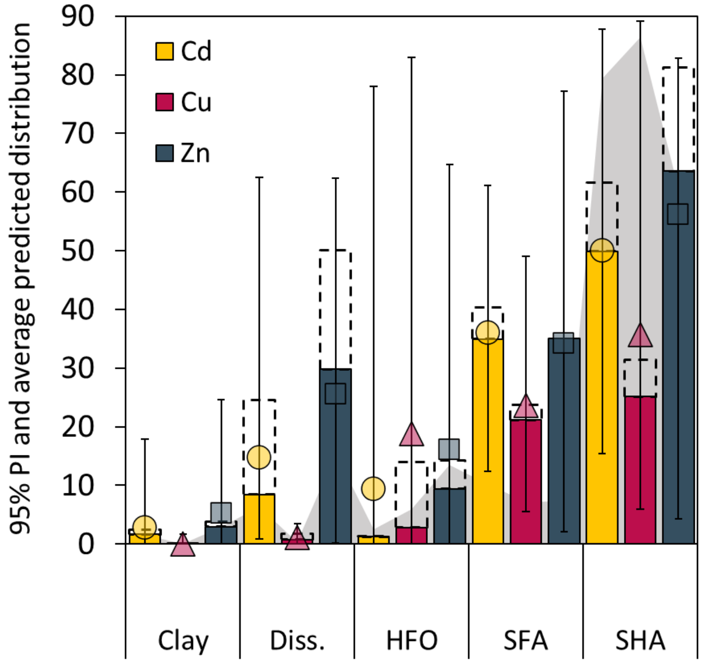

Figure S2.7. Uncertainty in predicted speciation (in percentage of HNO_3 total) quantified with the 95% prediction interval (PI) of all soils in the local scenario: median (filled bars), average (symbols), minimum and maximum (error bars). The shaded area in the background is the average speciation of all soils. The dashed bars represent the median PI in the global scenario.

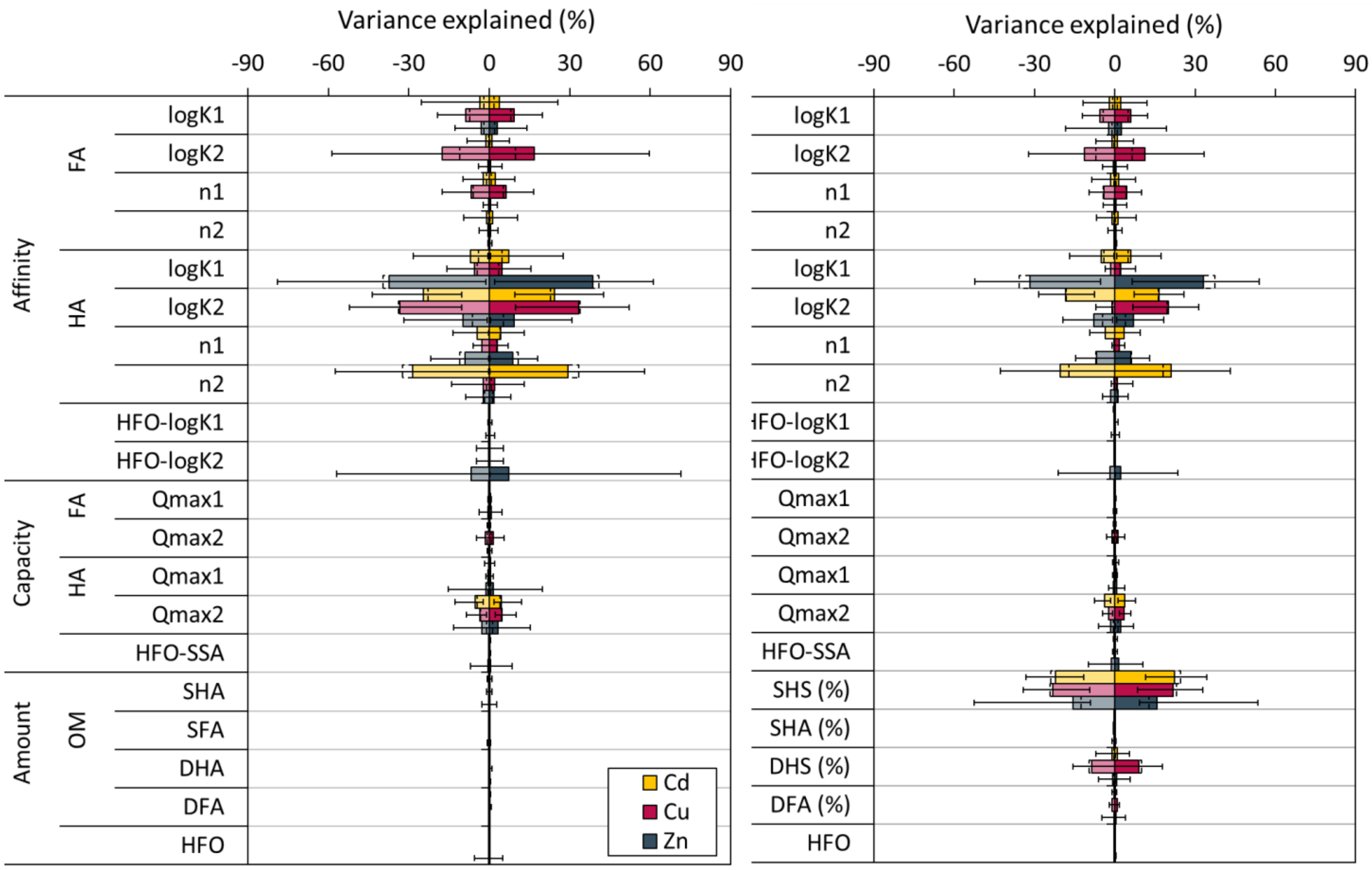

Figure S2.8. Sensitivity of predicted dissolved element concentrations to model parameters and input values in the local (left graph) and global (right graph) uncertainty analysis. The Top Marginal Variance (TMV) is also given in the main text (Figure 5). Here, the Bottom Marginal Variance (BMV) is included (given as negative values for ease of visualization). For each parameter the average (filled bar), median (dotted bar), minimum and maximum (error bars) of all soils are shown.

The influence of soil properties

To gain insight into the soil-dependency of model prediction uncertainty, we related soil properties the IQR. We considered pH, SOM and HNO_3-reactive total element (M_{react}) as the relevant soil properties. Normality was tested with the Jarque-Bera test and values were log-transformed where necessary. A forward stepwise regression selection was done based on the F-statistic with single effects only. Upon finding the optimal model, a two-sided t-test was carried out to establish the significance of model coefficients ($p < 0.05$). The prediction uncertainty was found to decrease with increasing SOM and M_{react} for all species (Table S2.1). The predicted dissolved concentration of cadmium and zinc also became less uncertain with pH. Important results are further discussed in the main text.

Table S2.1. The effect of soil properties on the uncertainty of the predicted speciation of Cd (■), Cu (■) and Zn (■), as indicated by the variables selected in the linear model predicting the (IQR). The effect of soil properties on IQR can be (+), negative (-) or not significantly different from zero (ns).

**IQR**	**pH**			**M_{react}**			**SOM**		
Diss.	-		-	-	ns	-	-		-
Clay	-	-	-	-	-	-	-	-	-
HFO	+	+	+		-	-	-	-	-
SFA		+	-			-			-
SHA		+	-	-	-	-		-	ns

SI.3 Correlation matrices NICA parameters

The NICA-Donnan parameters were found to be correlated, as was also observed by Groenenberg et al. (4). For the uncertainty analysis, correlations between parameters were imposed when sampling the parameters from their quantified uncertainties. Here, we used the correlation matrices that emerged during the PEST-ORCHESTRA (5) fitting procedure (Table S3.1). For Cu and Cd, the matrices were taken from the generic fit, i.e. when all available data per element were fitted at once. For Zn there was one dataset per humic substance that had the best fit, and from which the fitted parameters were used in the uncertainty analysis (see SI.7). The correlation matrices differed strongly per element, and Zn generally had higher correlation coefficients than Cd and Cu.

Correlation coefficients were applied per metal by first drawing a random sample from a probability distribution (values between 0-1), such that the same distribution per $logK_i$ and n_i was obtained. During this step, the *mvrnorm* function from the *MASS* package (version 7.3-61) allowed for imposing the specified covariance matrix (6). Subsequently, the probability distribution of each parameter was transformed with the *qtruncnorm* from the *truncnorm* package (version 1.0-9) to its normal distribution based on the average, standard deviation and minimum and maximum values (due to truncating) that was obtained within this study (SI.7 and SI.8) (7).

Table S3.1. Correlation matrices obtained from PEST-ORCHESTRA.

		Humic acid			Fulvic acid		
		**$logK_2$**	**n_1**	**n_2**	**$logK_2$**	**n_1**	**n_2**
**Cu**	**$logK_1$**	0.03	0.41	-0.53	0.43	0.85	-0.41
	**$logK_2$**		0.49	-0.54		0.28	-0.92
	**n_1**			-0.34			-0.12
**Cd**	**$logK_1$**	-0.11	0.93	-0.70	0.25	0.96	-0.40
	**$logK_2$**		0.14	0.03		0.42	0.19
	**n_1**			-0.75			-0.31
**Zn**	**$logK_1$**	0.72	0.91	-0.74	-0.39	0.99	-0.76
	**$logK_2$**		0.80	-0.92		-0.27	0.62
	**n_1**			-0.67			-0.72

In the main article, the model uncertainty was studied with the correlation matrices from Table S3.1. However, the model sensitivity was studied without correlation matrices to make sure that the parameters were sampled statistically independent. For completeness, we here briefly explain the effect of this choice on model accuracy and uncertainty. Without correlation matrices the uncertainties (IQR and 95% prediction intervals) remained similar or became larger for all three elements, especially for the amount bound to solid humic and fulvic acid (compare Figure S3.1 with Figure S2.7 and Figure 4 in the main text). This seems to suggest that the contrasting element-specific correlations between parameters are valuable, and we consider the PEST-ORCHESTRA correlations matrices are the most realistic to date, although future research could better establish these values and their mechanistic basis.

If we would have carried out the sensitivity analysis with the correlation matrices that were used in the uncertainty analysis, we would obtain the sensitivity profile of Figure S3.2 (compare with Figure S2.8 for the situation without correlation matrices). It can be seen that BMV values for NICA affinity parameters are much smaller than TMV values, which can be explained by the correlations between the parameters: the TMV is calculated as the variance explained by one variable, but the BMV is the variance not explained by excluding that variable from the full model with all variables. Because of the high correlations between parameters, the variance explained by one variable can be explained almost equally with another variable, thus reducing the BMV. Nevertheless, the general result is the same with or without correlations: the HA affinity parameters are most influential for model prediction uncertainty for all three elements, and for Cu there is also sensitivity to FA affinity parameters. Moreover, in the global scenario the assumption on the amount of solid OM that is reactive remained an influential variable with and without correlations between parameters.

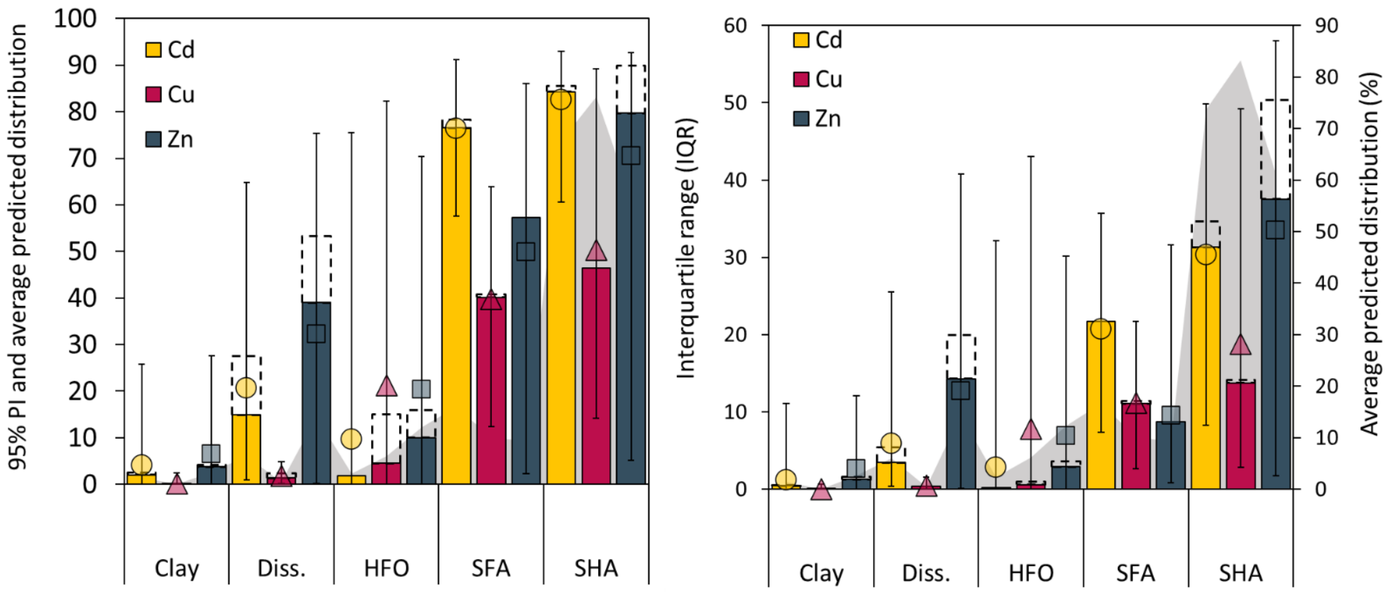

Figure S3.1. Uncertainty in predicted speciation (in percentage of HNO_3 total) quantified with the 95% prediction interval (PI) on the left, and the interquartile range (IQR) on the right. The values are obtained for the local scenario, sampling with no correlation between NICA parameters, and as such they supplement Figure S2.7 and Figure 4 (main text) where the same analysis was done but with the element-specific correlation coefficients established in Table S3.1. Values are for all soils in the local scenario: median (filled bars), average (symbols), minimum and maximum (error bars). The shaded area in the background is the average speciation of all soils. The dashed bars represent the median PI in the global scenario.

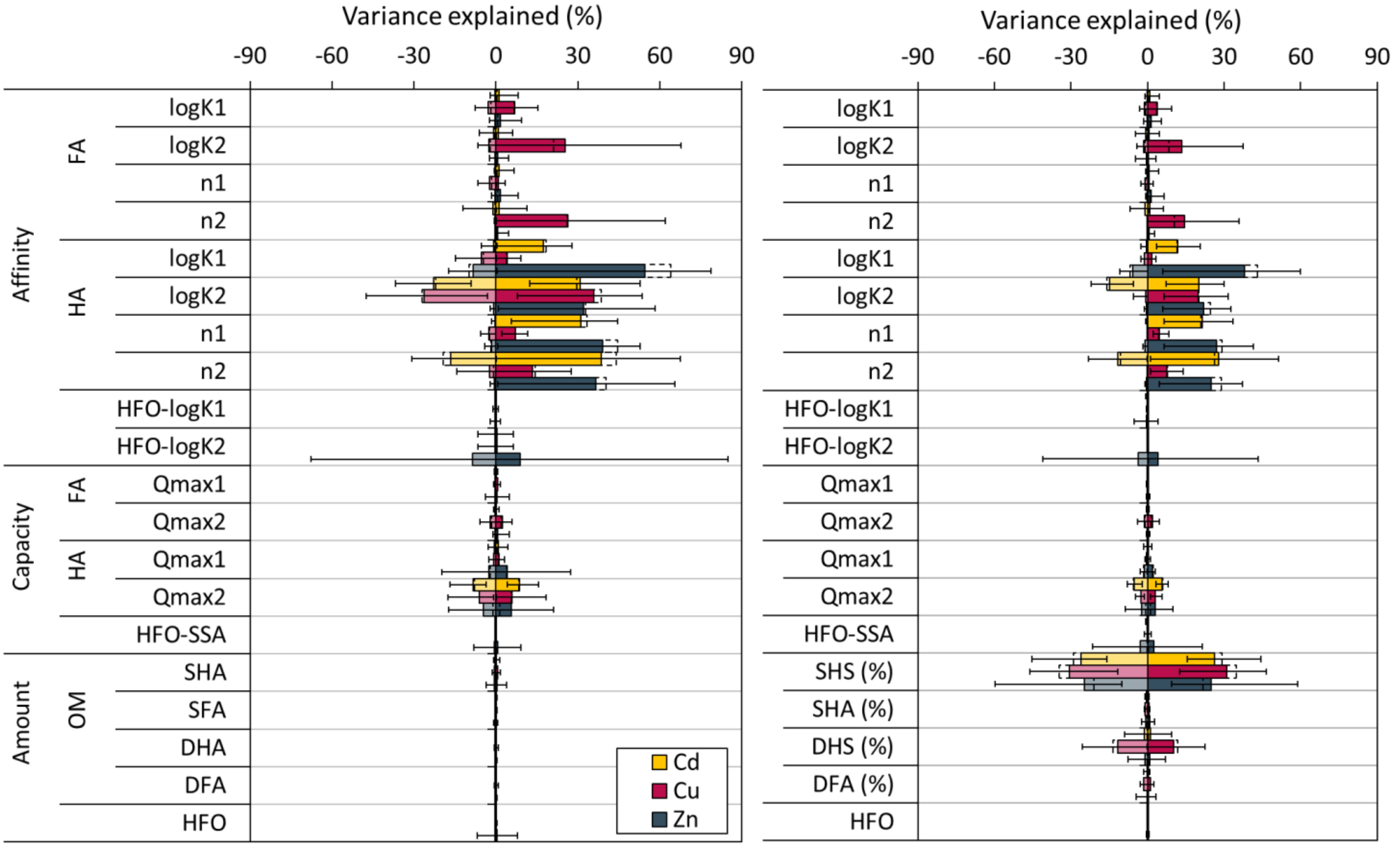

Figure S3.2. Sensitivity of predicted dissolved element concentrations to model parameters and input values in the local (left graph) and global (right graph) uncertainty analysis. Given are the Top Marginal Variance (TMV; right part of each graph) the Bottom Marginal Variance (BMV; left part of each graph, given as negative values for ease of visualization). The values are obtained for the local scenario, sampling with no correlation between NICA parameters, and as such they supplement Figure S2.8 where the same analysis was done but with the element-specific correlation coefficients established in Table S3.1. For each parameter the average (filled bar), median (dotted bar), minimum and maximum (error bars) of all soils are shown.

SI.4 Linear free energy relationships (LFERs)

4.1 Comparison LFER methods

Linear free energy relationships (LFERs) are useful where limited datasets are available of binding, such as for of Al, Fe and Mn. There are multiple ways ways to quantify LFERs, and we compared two of them. First, Tipping et al. (8) applied relationships to estimate model parameters. They used the comparison between metal-ligand and proton-ligand binding for this, with the relationship formulated by Irving and Rossotti (1956): $\log K_{ML} = \alpha \log K_{HL} \quad \beta$. The analysis showed that regression could be performed at fixed intercept at zero, leaving the α_O as the element-specific *Irving-Rossotti slope* that is derived for monodentate binding to negatively-charged oxygen donor atoms (i.e. including carboxylic and phenolic groups). Since α_O becomes the ratio between $\log K_{HL}$ and $\log K_{ML}$ it means that if a metal binds relatively strongly to these ligands compared to protons, they must also have a relatively high logK for binding to humic substances that contain these ligands. The older Model VI describes metal binding to two types of surface groups (A and B, mostly consisting of carboxylic and phenolic groups, respectively) via average binding constants $\log K_{MA}$ and $\log K_{MB}$ and spreads around this average for the four groups per type (ΔLK_{A1} and ΔLK_{B1}). In the subsequent Model VII it was found that $\log K_{MA}$ could be related to $\log K_{MB}$ via the ratio of the proton binding equilibrium constants, and the spread factors could be eliminated completely. Hence, only $\log K_{MA}$ was related to α_O with an R^2 of 0.68 and 0.80 for HA and FA, respectively.

Alternatively, Milne et al. (2) used the hydrolysis constant (K_{OH}). The hydrolysis constant is the ease with which a free metal ion (e.g. M^{2+}) interacts with water: $K_{OH} = [MOH^+][H^+]/[M^{2+}]$. For the binding constant the $\log K_{ML} = [ML]/[M^{2+}][L]$, the idea is that when an element is easily hydrolysed, it also binds easier to functional groups (i.e. $\log K_{OH}$ is positively related to $\log K_{ML}$). Overall, the Milne approach can be characterized as 'from the perspective of the element' (i.e.

easily hydrolysed means easily bound to humic substances), whereas the Tipping approach has 'the perspective of the ligand' (i.e. elements that bind relatively strongly to ligands compared to H^+ also bind strongly to humic substances). We compared both methods and there were few indications that either one performs better, except when comparing R^2 values the relationship with $\log K_1$ for FA, which was 0.49 with K_{OH} and 0.15 for IR (no further results shown). Based on this, and for consistency with the Milne et al. (2) generic database, we decided to use the hydrolysis constant to construct the LFERs.

For the derivation of the LFERs (Table S4.1) we initially used all parameters that were fitted independently by Milne et al. (2). It was observed that the relationship between K_{OH} and n_1 was very similar for HA and FA, hence data were combined to derive a single LFER for n_1 (the same was done by Milne et al. (2)). The FA n_1 parameters for Fe (value 0.30) was considered an outlier and removed (Figure S4.1). The n_2 was based on n_1 with the same formula given in Milne et al. (2): $n_2 = 0.76*n_1$ where the factor of 0.76 ± 0.17 (standard deviation, n=12).

We observed that linear $\log K_i$-$\log K_{OH}$ relationships accurately described the data, and that the difference with the exponential relationship $\log K_i^{ni}$-$\log K_{OH}$ as used by Milne et al. (2) is small in the range of $\log K_{OH}$ values of the elements that could be fitted freely based on adsorption data (Figure S4.2). Nevertheless, the approach by Milne et al. (2) would lead to strong deviations outside this $\log K_{OH}$ range, such as for Fe(III) adsorption to FA which was estimated to be described with a $\log K_1$ of 6.0 and a $\log K_2$ of 36 by Milne et al. (2). Whereas, the study of Hiemstra et al. (9) found fitted values of 2.70 and 8.30, respectively. The estimated values with our (linear) approach were 1.5 and 19.4, respectively; although this is still a substantial deviation, our estimates are much closer to the observed values than the estimates of Milne et al. (2). Based on the above, we derived LFERs for $\log K_i$ instead of $n_i \log K_i$ as Milne et al. (2) did. To derive our LFERs, for FA the Zn and Al $\log K_1$ were considered outliers and removed, similarly the Al $\log K_1$ for HA was removed (Figure S4.2).

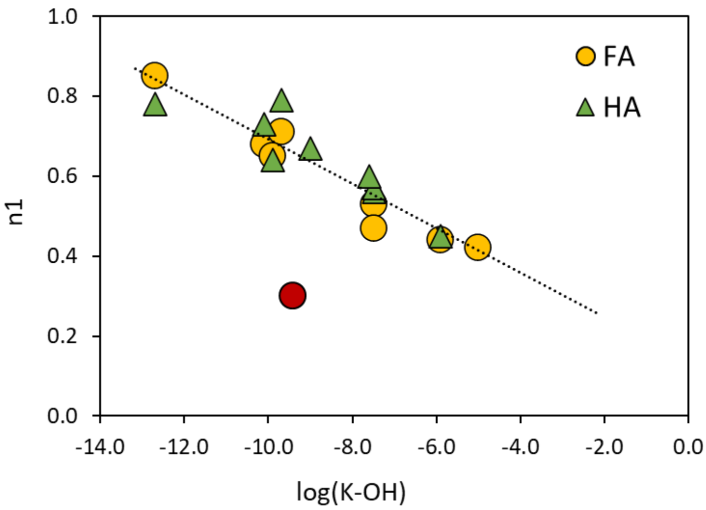

Figure S4.1. Relationship between K_{OH} and n_1. Regression line parameters given in Table S.4.1.

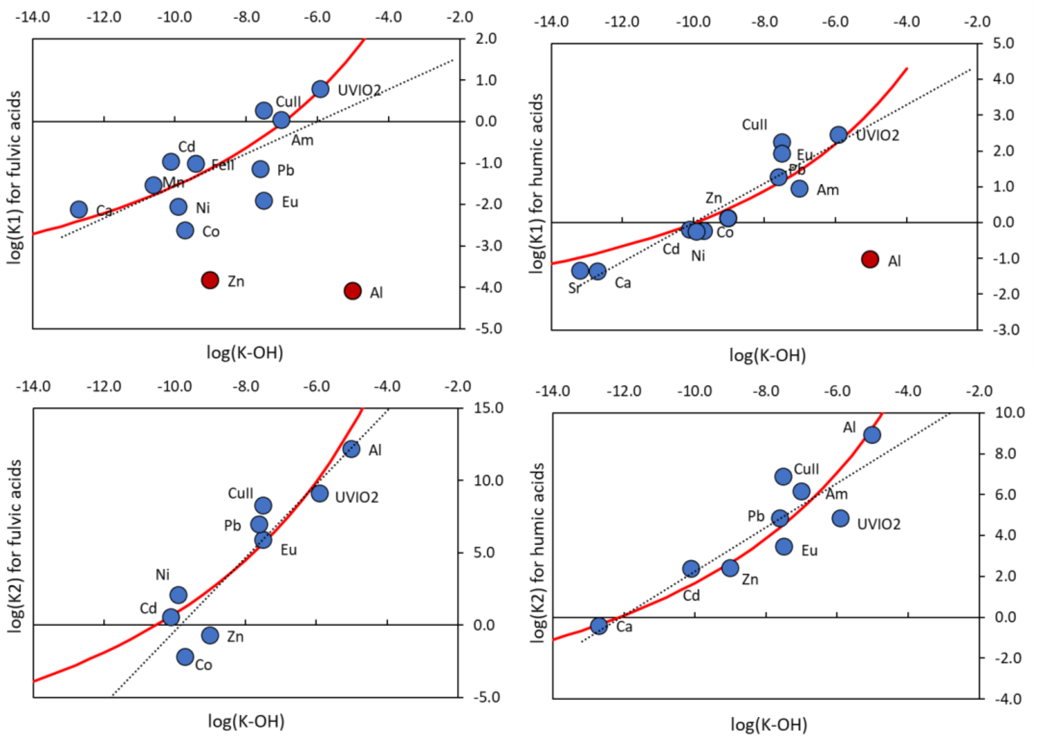

Figure S4.2. Linear relationships between K_{OH} and $\log K_i$ parameters for fulvic and humic acids. Regression equation (dotted line) parameters are given in Table S4.1. The red line depicts the $\log K_i$ values that would be calculated with the exponential LFERs used by Milne et al. (2).

Table S4.1. Regression parameters of linear free energy relationships (LFER) and application of these formulas to estimate binding parameters for Al, Fe and Mn. For FA-Fe(III) we used the parameters (in italics) derived by Hiemstra et al. (9).

Linear free energy relations (LFERs)

		$\log K_1$	$\log K_2$	n_1	n_2
**HA**	#	11	9	17	$0.76 \cdot n_1$
	**Slope**	0.55	1.08	-0.06	
	**Int.**	5.48	13.0	0.14	
	R^2	0.88	0.80	0.87	
**FA**	#	12	9	FA=HA	
	**Slope**	0.39	2.53		
	**Int.**	2.34	25.0		
	R^2	0.49	0.85		

Estimations

		$\log K_{OH}$	$\log K_1$	$\log K_2$	n_1	n_2
**Al**	**HA**	-5.0	2.74	7.64	0.41	0.31
	**FA**		0.39	12.3		
**Fe(III)**	**HA**	-2.2	4.27	10.7	0.26	0.20
	**FA***		*2.70*	*8.30*	*0.36*	*0.23*
**Mn**	**HA**	-10.6	-0.33	1.59	0.72	0.55
	**FA**		-1.79	-1.88		

4.2 Aluminum parameters

Our LFER parameters for Al differ substantially from those fitted by Milne et al. (2), especially regarding a higher $\log K_1$ for both HA and FA. As mentioned in the manuscript, our parameters describe the binding to isolated HA slightly better, and to FA slightly worse, compared to the Milne et al. (2) parameters (with the accuracy being in general relatively low for both humic substances compared to other elements). Here, we elaborate on our decision to present our LFER-Al parameters as the new generic parameters. Future studies can improve Al parameters by generating more adsorption data and by reconsidering the Donnan model, as discussed below.

For FA we fitted together the four datasets of Al binding to isolated fulvic acids used by Tipping et al. (8). The Milne et al. (2) parameters performed slightly better than our LFER parameters considering the RMSE (0.41 vs. 0.56) and ME (0.10 vs. 0.35). However, with the Milne et al. (2) parameters on average 64% of FA-bound Al was predicted to be present in the Donnan volume (median 83%, range 0.0-97%). In comparison, the average with our LFER parameters was 19% (median 19%, range 0.0-63%). Hence, the Milne et al. (2) parameters could describe the binding of Al to FA with relatively low specific binding ($\log K_1$) because a large share of Al was electrostatically bound. Our LFER parameters had stronger specific binding (higher $\log K_1$) and less electrostatically bound Al. The difference between the total amount of Al predicted to be bound to FA with our LFER parameters and with Milne et al. (2) parameters was especially large at higher ionic strength (IS, 0.01 M), pH ~4.0-5.5 and median free Al activities (pAl ~6-10) (Figure S4.3). Under such conditions, specific binding is less due to protonation of functional groups and the Donnan volume is relatively small with a correspondingly high concentration of negative charge, both of which favor electrostatic binding especially of trivalent cations such as Al^{3+} in the Donnan phase.

Whereas the Milne et al. (2) Al parameters can describe binding to isolated FA slightly better than our LFER parameters (keeping in mind the generally low accuracy), they are expected to underperform in soil applications because binding is disproportionally assigned to the Donnan volume. Cations such as Cd^{2+}, Cu^{2+} and Zn^{2+} are expected to bind mostly specifically to FA functional groups, and to describe such binding accurately it is important to include competition of cations such as Al^{3+}. With the Milne et al. (2) parameters competition would be relatively limited compared to with our LFER parameters (mostly due to the low $\log K_1$). This could explain in part why in our work multi-surface model predictions of dissolved Cd, Cu and Zn were better with our LFER Al-parameters than with the Milne et al. (2) Al parameters (RMSE 0.02-0.08 log-units lower, data not shown). We thus present our LFER parameters for Al to meet the recognized need for stronger competition of Al for FA functional groups (10).

For HA we fitted together the datasets of Al binding to isolated humic acids used by Tipping et al. (8), and added the dataset by Weng et al. (11). We found that our LFER parameters outperformed those by Milne et al. (2) in terms of the RMSE (0.33 vs. 0.40) and ME (0.17 vs. -0.22) (Figure S4.4). Although similar issues could occur with the Donnan volume as for FA (especially given that most adsorption experiments were carried out at higher IS than for FA data), the amount of Al bound electrostatically was lower (on average 7% and 35% for our LFER parameters and Milne et al. (2) parameters, respectively). Additionally, differences between the predicted HA-bound Al between both parameter sets were less clearly related to pH and ionic strength, yet differences were larger in the same range of free Al (pAl ~6-7). Also for HA the binding of Al is described with a relatively low accuracy compared to other elements, yet our parameters yield better predictions and are thus presented new generic values. Such higher specific binding constants ($\log K_i$) have been found in literature to be necessary to accurately describe competition of Al with Cd and Pb (12) and with rare earth elements (10).

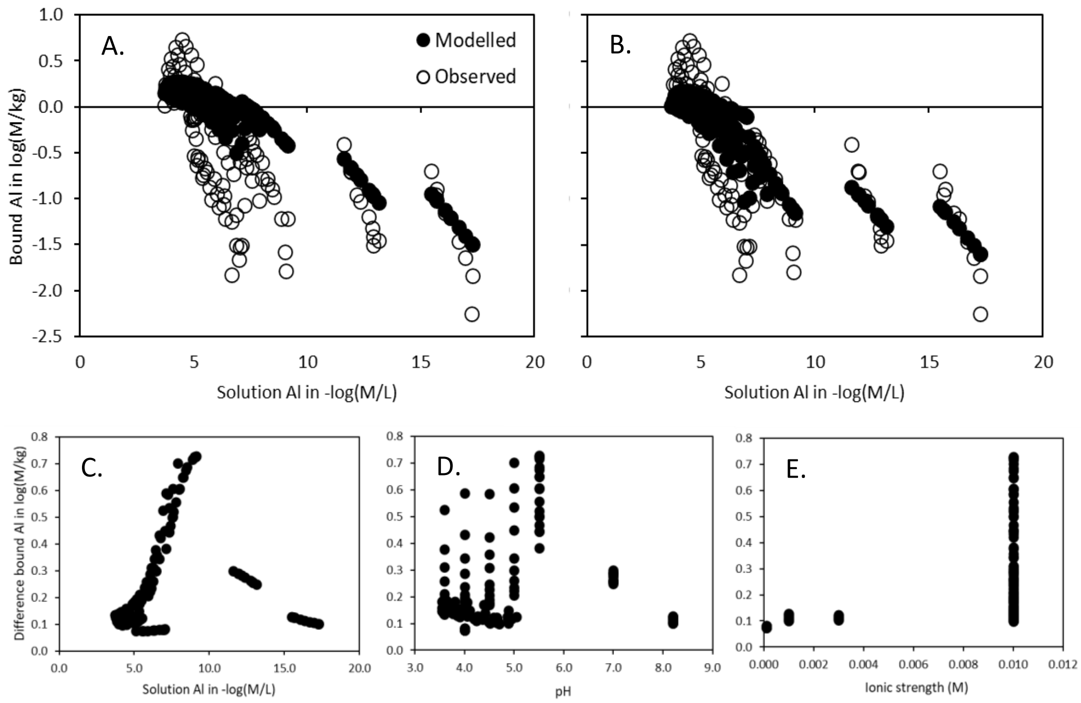

Figure S4.3. Binding of Al to FA observed and predicted with our LFER parameters (A) and with Milne et al. (2) (B). Lower graphs give the difference between modelled bound Al (A minus B) as a function of solution Al activity (C), pH (D) and ionic strength (E).

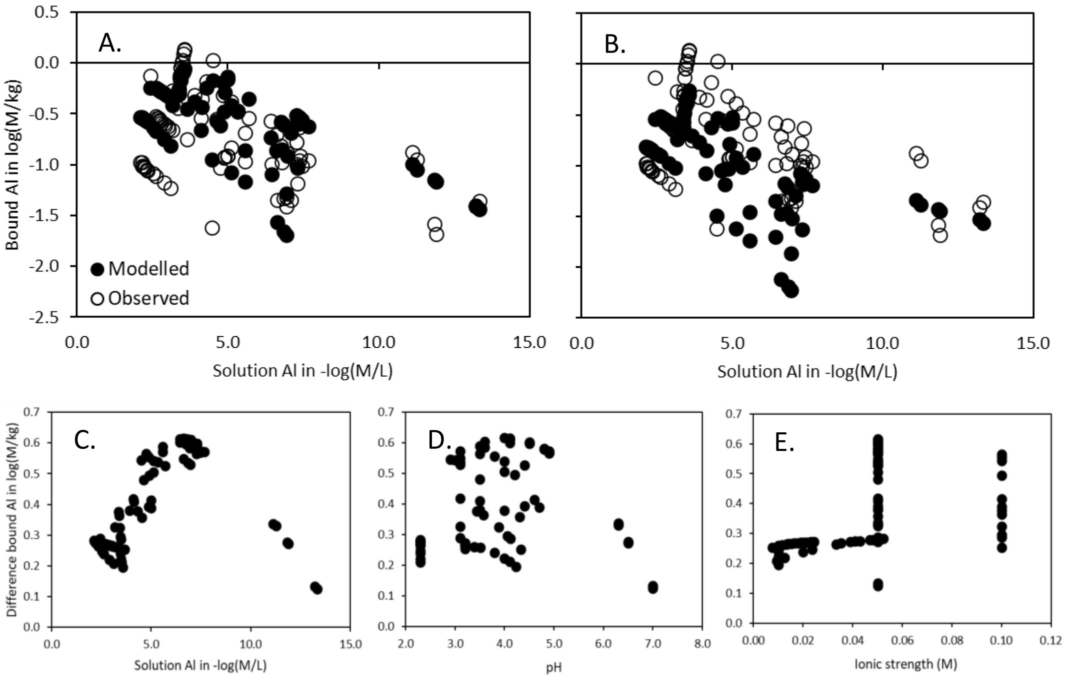

Figure S4.4. Binding of Al to HA observed and predicted with our LFER parameters (A) and with Milne et al. (2) (B). Lower graphs give the difference between modelled bound Al (A minus B) as a function of solution Al activity (C), pH (D) and ionic strength (E).

SI.5 Uncertainty in organic matter functional group density (Qmax)

For the uncertainty in Qmax values we used the data from Milne et al. (1), where in Table 2 the individual fits of proton binding datasets are given, including $Qmax_1$ and $Qmax_2$. To derive generic values, Milne et al. (1) used single semi-generic descriptions where all data for FA were fitted together, and HA data were also fitted together. Nevertheless, $Qmax_1$ and $Qmax_2$ were still allowed to vary per each individual dataset that formed part of the entire dataset. Next, the distribution parameters ($logK_i$, n_i etc.) found in this way were fixed and all data was refitted with these values to arrive at one generic value for $Qmax_1$ and $Qmax_2$ for FA and HA. Because of this approach, the average values from their Table 2 differ slightly from the generic values given in Table 4. In line with how we derived the uncertainty in the other model parameters, when calculating the average and standard deviation we only included individual datasets that could be fitted independently (i.e. without fixing part of the parameters). For the $Qmax_2$ there seemed to be two outliers (Figure S5.1). These values were removed because it brought the average value closer to the generic values (Table S5.1).

Table S5.1. Generic values, averages and standard deviations of Qmax values for fulvic and humic acids. See text for details.

		**Fulvic acid**		**Humic acid**	
		**Qmax$_1$**	**Qmax$_2$**	**Qmax$_1$**	**Qmax$_2$**
**Milne et al. (1)**	**Generic**	5.88	1.86	3.15	2.55
	**Average**	5.66 (n = 25)	2.57 (n = 18)	3.17 (n = 23)	2.66 (n = 20)
	**SD**	1.25	1.94	0.89	1.37
**Present study**	**Average**	5.30 (n = 14)	2.70 (n = 14)	3.25 (n = 13)	2.34 (n = 13)
	**SD**	1.26	2.18	0.97	1.43
without outliers	**Average**	*Idem.*	1.96 (n = 12)	*Idem.*	*Idem.*
	**SD**		1.16		

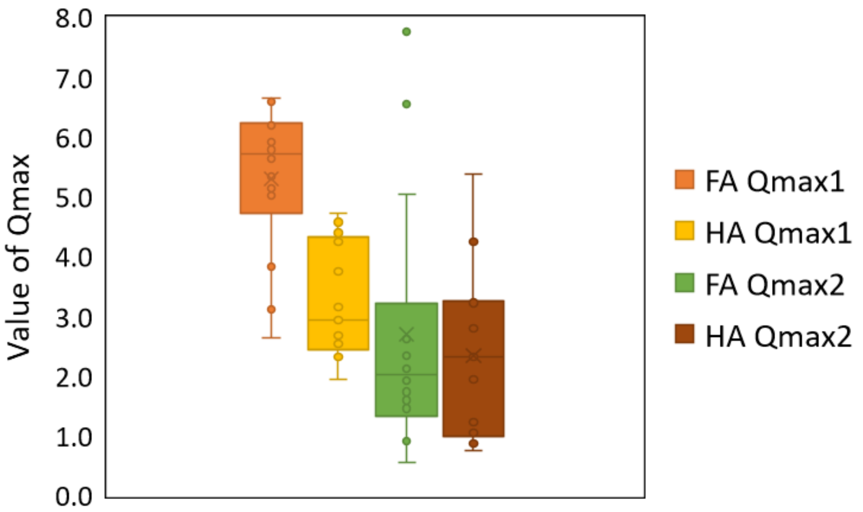

Figure S5.1. Distribution of Qmax values for humic and fulvic acids given in Milne et al. (1).

SI.6 Uncertainty in metal-oxide binding parameters

To quantify the uncertainty in HFO binding constants we revisited the fits of individual datasets performed by Dzombak and Morel (13). They fitted individual datasets and derived best-estimate (i.e. generic) values based on weighting the individual predictions with the inverse of their standard deviation. In line with our approach to define the uncertainty in NICA-Donnan parameters, we calculated the average and standard deviation without weighting. This can in part be justified as a higher standard deviation for individual fits does not necessarily mean that the binding parameters are more uncertain. We obtained the data from the tables published for zinc (Table 6.9), cadmium (Table 6.14) and copper (Table 6.23).

The GTLM for HFO considers *strong* and *weak* complexes that can be formed, which binding constants are indicated with $\log K_1$ and $\log K_2$, respectively (Table S6.1). For Cu no $\log K_2$ values could be fitted, and for Cd only one individual database could describe $\log K_2$. Nevertheless, the latter had a high overall variance (defined as the weighted sum of squares of the residuals divided by the degrees of freedom; this value should be as small as possible, and was regularly around 1.0, but for this fit was 23.1). Additionally, the $\log K_1$ for this particular dataset can be considered an outlier compared to all other fits, with a value of -0.51 whereas all others (n = 23) were between 0.08-1.08, which seems to suggest the $\log K_2$ value for Cd is highly uncertain. The dataset 1ECD22 for Cd was removed when calculating the average.

Because Cd was based on one relatively poor fit and Cu derived from LFERs we applied a higher uncertainty to these values (Table S6.1). For Zn it was observed that the minimum and maximum $\log K_2$ were about 1 log-unit apart (-2.23 to -1.18). Hence we took the best estimate values given above as the average and sampled from a uniform distribution between -1 and +1 log-units of this value, i.e. between -3.90 and -1.90 for Cd and -0.4 and 1.6 for Cu.

Table S6.1. Affinity parameters for metal binding to HFO. The generic values are those derived by Dzombak and Morel (13), and averages and standard deviations (SD) are calculated by individual fits given in the same reference.

Parameter	Element	No.	Generic	Average	SD
**$logK_1$**	Zn	21	0.99	0.87	0.28
	Cd	23	0.47	0.49	0.23
	Cu	10	2.89	2.90	0.29
**$logK_2$**	Zn	7	-1.99	-1.82	0.34
	Cd	1	-2.90	[-]	[-]
	Cu	0	0.6	[-]	[-]

SI.7 Annotated summary of individual NICA-Donnan database fits

The tables below give the fits of individual data-sets (Table S7.1), with comments of fitting choices that were made to obtain adequate fits. For each element-humic substance we first give the fitting boundary conditions (start value, lower bound and upper bound). Next, a summary is given of (i) the values reported by Milne et al. (2), (ii) The values obtained by fitting all data used by Tipping et al. (8), (iii) the values obtained by fitting all data, including additional databases published after the publication of Tipping et al. (8), and (iv) the average and standard deviation of the individual datasets that could be fitted. Finally, the fits per datasets are given, where the coding follows that of Milne et al. (2), which was also continued in Tipping et al. (8). Values marked red have reached the boundary conditions, indicating that the dataset could not be fitted adequately. Datasets where at least one value was red were not used in the calculation of the average and standard deviation. When a dataset could not be fitted, the data was inspected and at times an adequate fit could be obtained by removing e.g. several clear outliers.

As mentioned in the main text, only one dataset per humic substance was used for Zn. For HA (Table S7.6), HZn3 was the only database that could be fitted individually within the boundary conditions. Fitting all data together lead to a similar $\log K_1$, but the $\log K_2$ was about four log-units lower. Comparing this with the $\log K_2$ obtained from the LFER (SI.4), where the value was 3.32, we concluded that the HZn3 dataset individually was a better description of Zn adsorption behaviour to HA compared to all data together, because $\log K_2$ should in general be substantially higher than $\log K_1$, and the values are more in line with LFER predictions. Therefore, we used the HZn3 parameters as generic. Similarly, for FA (Table S7.7) we found that only FZn5 could be fitted individually, and that this fit was better than fitting all data together, which could not be fitted within the boundary conditions and would divert to unrealistic values. Therefore, we used FZn5 parameters as the generic values.

Table S7.1. Codes for individual datasets fitted with PEST-ORCHESTRA.

Element	HS	Code	Reference
**Cu**	**HA**	HCu_01 to HCu_14	See Tipping et al. (8) and Milne et al. (2)
		HCu_15	Marang et al. (14)
		HCu_16	Vidali et al. (15) – GHA
		HCu_17	Vidali et al. (15) – PPHA
		HCu_18	Xu et al. (16) – JGHA
		HCu_19	Xu et al. (16) – JLHA
		HCu_20	Xu et al. (16) – PAHA
	**FA**	FCu_01 to FCu_16	See Tipping et al. (8) and Milne et al. (2)
		FCu_17	Wang et al. (17)
		FCu_18	Xu et al. (16)
**Cd**	**HA**	HCd_01 to HCd_10	See Tipping et al. (8) and Milne et al. (2)
		HCd_11	Oste et al. (18)
		HCd_12	Ratie et al. (19)
	**FA**	FCd_01 to FCd_06	See Tipping et al. (8) and Milne et al. (2)
		FCd_07	Janot et al. (5)
**Zn**	**HA**	HZn_01 to HZn_03	See Tipping et al. (8) and Milne et al. (2)
		HZn_04	Jouvin et al. (20)
	**FA**	FZn_01 to FZn_04	See Tipping et al. (8) and Milne et al. (2)
		FZn_05	Janot et al. (5)

Table S7.2. Copper binding to humic acid. See text appendix SI.7 above for more information.

Copper to humic acid						
Data	#	$logK_1$	n_1	$logK_2$	n_2	Comments
Fitting boundary conditions						
Start	NA	2.23	0.56	6.85	0.34	[-]
Lower	NA	-4.0	0.1	1.0	0.1	[-]
Upper	NA	8.0	0.9	12	0.9	[-]
Summary						
Milne (2003)	822	2.230	0.56	6.850	0.34	Values given in Milne et al. (2003).
Milne	822	2.280	0.56	6.750	0.31	Data of Milne et al. (2003) fitted again.
Tipping	945	2.298	0.54	6.727	0.33	Data of Tipping (incl. Milne)
Wiersma	1374	2.149	0.53	7.218	0.36	Data of Wiersma (incl. Milne and Tipping)
Average ± stand. dev.	[16]	1.691 ±0.629	0.49 ±0.08	7.080 ±1.353	0.42 ±0.15	Average of datasets that fitted within the boundary conditions (i.e. excluding those datasets that have at least one value in red below).
Milne et al. (2003)						
HCu1-a	64	2.334	0.43	4.279	0.9	The same dataset as used by (a) Milne et al. (2003) and (b) Tipping et al. (2011).
HCu1-b	40	2.375	0.41	5.581	0.67	
HCu2	Not used					Data not used by Tipping et al. (2011).
HCu3	161	2.587	0.57	6.413	0.47	[-]
HCu4-a	115	0.471	0.51	7.196	0.36	The same dataset as used by (a) Milne et al. (2003) and (b) Tipping et al. (2011).
HCu4-b	102	0.481	0.48	7.088	0.33	
HCu5-a	25	0.798	0.46	3.741	0.9	The same dataset as used by (a) Milne et al. (2003) and (b) Tipping et al. (2011).
HCu5-b	17	1.149	0.69	10.168	0.19	
HCu6	18	2.164	0.58	6.794	0.23	[-]
HCu7	Not used					Data not used by Tipping et al. (2011).
HCu8	303	2.290	0.42	5.213	0.67	[-]

HCu9	51	1.598	0.53	7.338	0.39	[-]
HCu10	85	0.931	0.46	6.057	0.50	[-]
Tipping et al. (2011)						
HCu11	56	1.245	0.46	7.246	0.43	[-]
HCu12	6	-0.823	0.9	6.854	0.55	Not refitted because of the small dataset.
HCu13	39	1.422	0.41	6.841	0.32	[-]
HCu14	67	2.704	0.39	5.408	0.70	[-]
Wiersma et al. (2023)						
HCu15	12	-4	0.9	1	0.1	All parameters reached the boundaries. Data were not refitted with different start-conditions because of the small dataset.
HCu16-a	94	-0.029	0.1	7.586	0.45	There were five datapoints that visually deviated a lot from the adsorption curves. These were removed to yield a fit that is in line with the other values and does not reach the set boundaries.
HCu16-b	89	1.454	0.42	9.592	0.29	
HCu17-a	167	-1.086	0.1	8.440	0.28	Essentially two datasets, one an adsorption curve and the other a pH-dependence curve. The data for the latter was given as % bound, which does not necessarily correspond to free Cd^{2+}. These data (n = 14) were left out.
HCu17-b	153	2.121	0.47	8.001	0.36	
HCu18	62	1.699	0.50	7.326	0.36	These three datasets come from the same reference. They are humic acids from different sources hence fitted individually.
HCu19	61	1.265	0.50	7.570	0.37	
HCu20	64	1.573	0.47	6.640	0.45	

Table S7.3. Copper binding to fulvic acid. See text appendix SI.7 above for more information.

Copper to fulvic acid						
Data	#	$logK_1$	n_1	$logK_2$	n_2	Comments
Fitting boundary conditions						
Start	NA	0.26	0.53	8.26	0.36	[-]
Lower	NA	-6.0	0.1	2.0	0.1	[-]
Upper	NA	6.0	0.9	14	0.9	[-]
Summary						
Milne (2003)	541	0.26	0.53	8.26	0.36	Values given in Milne et al. (2003).
Tipping	671	-0.666	0.50	9.333	0.33	Data of Tipping (incl. Milne)
Wiersma	784	-0.674	0.51	9.236	0.34	Data of Wiersma (incl. Milne and Tipping)
Average ± stand. dev.	[13]	-0.765 ±0.975	0.51 ±0.10	7.218 ±1.206	0.47 ±0.13	Average of datasets that fitted within the boundary conditions (i.e. excluding those datasets that have at least one value in red below).
Milne et al. (2003)						
FCu1	33	-0.007	0.72	6.892	0.58	[-]
FCu2-a	22	-4.600	0.9	8.606	0.49	Changed boundary conditions closer to the average found for all other datasets (logK1 -1.0; logK2 8.0; n1 and n2 0.4). This yield a fit that was within the boundary conditions. However, the fits did depend strongly on small changes in boundary conditions, e.g. at logK2 7.0 the fit was inadequate.
FCu2-b	22	-1.157	0.46	7.049	0.50	
FCu3	60	0.027	0.42	8.974	0.29	[-]
FCu4	Not used					Only two datapoints.
FCu5	27	Does not reach fit				Changing boundary conditions or using only part of the data did not yield a fit.
FCu6	24	-2.758	0.33	7.374	0.49	[-]
FCu7	15	-4.796	0.24	2	0.1	Tried to fit with different boundary conditions and removing some observations that seemed (visually) to

						deviate from the adsorption curve), but this did not improve the fit.
FCu8	17	-0.486	0.63	5.084	0.81	[-]
FCu9	15	-0.219	0.38	2	0.1	Tried to fit with different boundary conditions and removing some observations that seemed (visually) to deviate from the adsorption curve), but this did not improve the fit.
FCu10	60	-0.107	0.51	8.217	0.43	[-]
FCu11	56	0.418	0.55	7.726	0.42	[-]
Tipping et al. (2011)						
FCu12	51	-3.708	0.9	8.518	0.46	Changing boundary conditions or fitting only one ionic strength (0.001 or 0.1 M) did not lead to better fits.
FCu13	63	0.436	0.48	8.582	0.42	[-]
FCu14	89	-0.684	0.62	8.223	0.37	[-]
FCu15	106	-0.784	0.57	6.902	0.44	[-]
FCu16	31	-1.506	0.50	6.238	0.47	[-]
Wiersma et al. (2023)						
FCu17	67	-2.181	0.47	7.460	0.54	[-]
FCu18-a	46	-1.147	0.42	2	0.30	n2 got 'stuck' at 0.30, while the lower limit was set at 0.10. When the model was forced to fit n2 > 0.31, it yielded an acceptable fit.
FCu18-b	46	-1.159	0.43	5.119	0.37	

Table S7.4. Cadmium binding to humic acid. See text appendix SI.7 for more information.

Cadmium to humic acid						
Data	#	$logK_1$	n_1	$logK_2$	n_2	Comments
Fitting boundary conditions						
Start	NA	-0.20	0.73	2.37	0.54	[-]
Lower	NA	-6.0	0.1	-4.0	0.1	[-]
Upper	NA	6.0	0.9	8.0	0.9	[-]
Summary						
Milne (2003)	518	-0.20	0.73	2.37	0.54	Values given in Milne et al. (2003).
Tipping	436	-0.611	0.63	2.196	0.61	Data of Tipping (incl. Milne)
Wiersma	455	-0.609	0.63	2.231	0.60	Data of Wiersma (incl. Milne and Tipping)
Average ± stand. dev.	[6]	-0.753 ±1.166	0.64 ±0.19	2.859 ±0.856	0.45 ±0.23	Average of datasets that fitted within the boundary conditions (i.e. excluding those datasets that have at least one value in red below).
Milne et al. (2003)						
HCd1	21	Unsure how to fit				In contrast to the other databases, the data are given as [base] (M) and total [Cd], instead of bound and free Cd.
HCd2	56	-1.748	0.47	2.321	0.84	Unlikely combination of n-values (n_2 higher than n_1) but logK's generally in line with other fits hence kept.
HCd3	192	-0.145	0.74	2.276	0.52	[-]
HCd4	10	-2.387	0.47	-4	0.9	Not refitted because of the small dataset.
HCd5	13	0.674	0.90	4.672	0.12	[-]
HCd6	14	0.093	0.9	-4	0.9	Not refitted because of the small dataset.
HCd7	54	-2.807	0.33	2.198	0.26	[-]
Tipping et al. (2011)						
HCd8	18	-6	0.9	3.009	0.61	Did not improve under different start and boundary conditions.

HCd9	28	-0.057	0.66	2.889	0.35	[-]
HCd10	51	-0.532	0.74	2.964	0.53	[-]
Wiersma et al. (2023)						
HCd11	19	-0.655	0.67	2.695	0.54	[-]
HCd12	149	0.797	0.69	1.098	0.9	In this dataset the Cd is given as %-adsorbed, hence no free Cd^{2+} data available but calculated assuming that all non-bound Cd was free. The logK's are also quite different from most other fit, hence this dataset can probably be excluded.

Table S7.5. Cadmium binding to fulvic acid. See text appendix SI.7 for more information.

Cadmium to fulvic acid						
Data	#	$logK_1$	n_1	$logK_2$	n_2	Comments
Fitting boundary conditions						
Start	NA	0.26	0.53	8.26	0.36	[-]
Lower	NA	-6.0	0.1	2.0	0.1	[-]
Upper	NA	6.0	0.9	14	0.9	[-]
Summary						
Milne (2003)	520	-0.97	0.68	0.50	*0.50*	Values given in Milne et al. (2003).
Tipping	280	-1.619	0.66	1.125	0.43	Data of Tipping (incl. Milne)
Wiersma	321	-1.644	0.66	1.224	0.54	Data of Wiersma (incl. Milne and Tipping)
Average ± stand. dev.	[5]	-2.588<br>±1.390	0.61<br>±0.14	2.488<br>±1.140	0.52<br>±0.28	Average of datasets that fitted within the boundary conditions (i.e. excluding those datasets that have at least one value in red below).
Milne et al. (2003)						
FCd1	33	-1.428	0.62	1.162	0.9	Data and adsorption curve look good, cannot find a reason why the fit would hit the boundary condition. The other three parameters are quite in line with other fits and the overall average.
FCd2	27	-0.678	0.71	3.134	0.47	[-]
FCd3-a	96	-6	0.9	4.978	0.45	The first dataset is those used by Tipping, the second the same dataset completely obtained from the original publication. Cannot find a reason why the first fit is off, as the adsorption curves look good.
FCd3-b	150	-4.593	0.52	1.670	0.49	
FCd4	70	-2.725	0.45	3.161	0.86	The n-values are not in line with expectations, as n_2 is generally lower than n_1, however removing these parameters does not change the averages and standard deviations much.

FCd5	10	-0.003	0.9	3.086	0.9	Did not refit under different conditions because of the limited observations.
Tipping et al. (2011)						
FCd6	44	-2.542	0.78	4.011	0.70	[-]
Wiersma et al. (2023)						
FCd7	41	-2.402	0.57	0.465	0.70	[-]

Table S7.6. Zinc binding to humic acid. See text appendix SI.7 above for more information.

Zinc to humic acid						
Data	#	$logK_1$	n_1	$logK_2$	n_2	Comments
Fitting boundary conditions						
Start	NA	0.11	0.7	2.39	0.5	[-]
Lower	NA	-6.0	0.1	-4.0	0.1	[-]
Upper	NA	6.0	0.9	8.0	0.9	[-]
Summary						
Milne (2003)	35	0.11	0.67	2.39	0.27	Values given in Milne et al. (2003).
Tipping	43	-0.062	0.57	0.932	0.46	Data of Tipping (incl. Milne)
Wiersma	54	-0.709	0.46	-0.123	0.18	Data of Wiersma (incl. Milne and Tipping)
Milne et al. (2003)						
HZn1-a	15	-3.412	0.1	2.770	0.9	Same dataset fitted freely (a) and with the nonideality parameters fixed at those of HZn3 (b). Fixing these parameters does not lead to logK's that could be reasonably expected.
HZn1-b	15	0.261	*0.59*	-2	*0.25*	
HCd2	Not used					Data not used by Tipping et al. (2011).
Tipping et al. (2011)						
HZn3	28	-0.251	0.59	4.087	0.25	[-]
Wiersma et al. (2023)						
HZn4-a	11	-5.487	0.9	-2	0.1	Same dataset fitted freely (a) and with the nonideality parameters fixed at those of HZn3 (b). Fixing these parameters does not lead to logK's that could be reasonably expected.
HZn4-b	11	-6	*0.59*	-2	*0.25*	

Table S7.7. Zinc binding to fulvic acid. See text appendix SI.7 above for more information.

Zinc to fulvic acid						
Data	#	$logK_1$	n_1	$logK_2$	n_2	Comments
Fitting boundary conditions						
Start	NA	-2.0	0.7	3.0	0.6	[-]
Lower	NA	-8.0	0.1	-4.0	0.1	[-]
Upper	NA	4.0	0.9	8.0	0.9	[-]
Summary						
Milne (2003)	25	-3.84	*0.67*	-0.73	*0.61*	Values given in Milne et al. (2003).
Wiersma	85	-0.710	0.89	-4	0.9	Data of Wiersma (incl. Milne and Tipping)
Milne et al. (2003)						
FZn1	2	Not fitted				Too few data points.
FZn2	7	Not fitted				Too few data points.
FZn3-a	19	-2.546	0.76	4.331	0.9	Same dataset fitted freely (a) and with the nonideality parameters fixed at those of FZn5 (b). logK's do not differ much between both fits, suggesting they are adequate and relatively independent of n-values.
FZn3-b	19	-1.973	*0.79*	2.928	*0.55*	
Tipping et al. (2011)						
FZn4-a	19	-1.398	0.74	-4	0.9	Same dataset fitted freely (a) and with the nonideality parameters fixed at those of FZn5 (b). logK's do not differ much between both fits, suggesting they are adequate and relatively independent of n-values.
FZn4-b	19	-1.216	*0.79*	-10	*0.55*	
Wiersma et al. (2023)						
FZn5	38	-1.292	0.79	2.428	0.55	[-]

SI.8 Summary sampling approach local and global uncertainty analysis

Table S8.1. Overview of sampling approach for model parameters and soil-specific input values. For OM we give the standard deviation (SD) with the average (Av.) as obtained by fitting individual datasets (our approach in the uncertainty and sensitivity analyses) and the SD obtained with the generic (Gen.) by PEST-ORCHESTRA for all data fitted together as reference. For Zn we used estimates of the SD (see manuscript for details), which are reported here.

**Affinity** parameters										
				**Local = Global sampling**						
				i* = *1			***i* = *2***			
				**Gen.**	**Av.**		**Gen.**	**Av.**		**Sampling**
OM	$\log K_i$	HA	Cu	2.15	1.69		7.22	7.08		Truncated normal distribution within two SD around mean.
			SD	0.03	0.63		0.10	1.35		
			Cd	-0.61	-0.75		2.23	2.86		
			SD	0.08	1.17		0.12	0.86		
			Zn	-0.25			4.09			
			SD	*1.5*			*1.5*			
		FA	Cu	-0.67	-0.77		9.24	7.22		
			SD	0.08	0.98		0.32	1.21		
			Cd	-1.64	-2.59		1.22	2.49		
			SD	0.22	1.39		2.92	1.41		
			Zn	-1.29			2.41			
			SD	*1.5*			*1.5*			
	n_i	HA	Cu	0.53	0.49		0.36	0.42		Truncated normal distribution within one SD around mean.
			SD	0.01	0.08		0.01	0.15		
			Cd	0.63	0.64		0.60	0.45		
			SD	0.02	0.19		0.05	0.23		
			Zn	0.59			0.24			
			SD	*0.2*			*0.2*			
		FA	Cu	0.51	0.51		0.34	0.47		
			SD	0.01	0.10		0.02	0.13		
			Cd	0.66	0.61		0.54	0.53		
			SD	0.05	0.14		1.91	0.28		
			Zn	0.79			0.54			
			SD	*0.2*			*0.2*			
HFO	$\log K_i$	Cu		2.89	2.90	0.29	0.6		[-]	Truncated normal distribution within two SD around mean.
		Cd		0.47	0.49	0.23	-2.90		[-]	
		Zn		0.99	0.87	0.28	-1.99	-1.82	0.34	
**Capacity** parameters										
OM	$Qmax_i$-FA			5.88	5.30	1.26	1.86	1.96	1.16	Truncated normal distribution within one SD around mean.
	$Qmax_i$-HA			3.15	3.25	0.97	2.55	2.34	1.43	

		**Local sampling**	**Global sampling**
HFO	SSA	Normal distribution with measured value and SD based on average CV found for all soils.	Truncated normal distribution within 1 SD around mean. Average ± SD was 614 ± 416.

**Amount** of surface			
OM	SHA<br>SFA	Normal distribution with measured value and SD based on CV of 5%.	Truncated normal distribution within 2 SD around mean. SOC being 50 ± 23% SHS and of this 74 ± 10% being SHA (remaining = SFA).
	DHA<br>DFA		Truncated normal distribution within 2 SD around mean. DOC being 30 ± 12% DHS and of this 96 ± 4% being DFA (one SD; remaining = DHA).
HFO			Same as in local analysis.

References

1. Milne CJ, Kinniburgh DG, Tipping E. Generic NICA-Donnan model parameters for proton binding by humic substances. Environ Sci Technol. 2001;35(10):2049-59.
2. Milne CJ, Kinniburgh DG, van Riemsdijk WH, Tipping E. Generic NICA-Donnan model parameters for metal-ion binding by humic substances. Environ Sci Technol. 2003;37(5):958-71.
3. Van Eynde E, Weng L, Comans RN. Boron speciation and extractability in temperate and tropical soils: A multi-surface modeling approach. Applied Geochemistry. 2020;123:104797.
4. Groenenberg JE, Koopmans GF, Comans RN. Uncertainty analysis of the nonideal competitive adsorption-donnan model: effects of dissolved organic matter variability on predicted metal speciation in soil solution. Environ Sci Technol. 2010;44(4):1340-6.
5. Janot N, Pinheiro JP, Botero WG, Meeussen JCL, Groenenberg JE. PEST-ORCHESTRA, a tool for optimising advanced ion-binding model parameters: derivation of NICA-Donnan model parameters for humic substances reactivity. Environmental Chemistry. 2017;14(1):31-8.
6. Venables W, Ripley B. Modern Applied Statistics with S, Springer, New York: ISBN 0-387-95457-0. 2002.
7. Geweke J, editor Efficient simulation from the multivariate normal and student-t distributions subject to linear constraints and the evaluation of constraint probabilities. Computing science and statistics: Proceedings of the 23rd symposium on the interface; 1991: Fairfax, Virginia: Interface Foundation of North America, Inc.
8. Tipping E, Lofts S, Sonke JE. Humic Ion-Binding Model VII: a revised parameterisation of cation-binding by humic substances. Environmental Chemistry. 2011;8(3):225-35.
9. Hiemstra T, van Riemsdijk WH. Biogeochemical speciation of Fe in ocean water. Marine Chemistry. 2006;102(3-4):181-97.
10. Otero-Fariña A, Janot N, Marsac R, Catrouillet C, Groenenberg JE, Lofts S. Rare earth elements binding humic acids: NICA–Donnan modelling. Environmental Chemistry. 2023;21(1).
11. Weng L, Temminghoff EJ, Van Riemsdijk WH. Aluminum speciation in natural waters: measurement using Donnan membrane technique and modeling using NICA-Donnan. Water Res. 2002;36(17):4215-26.
12. Pinheiro JP, Mota AM, Benedetti MF. Effect of Aluminum Competition on Lead and Cadmium Binding to Humic Acids at Variable Ionic Strength. Environmental Science & Technology. 2000;34(24):5137-43.
13. Dzombak DA, Morel FM. Surface complexation modeling: hydrous ferric oxide: John Wiley & Sons; 1990.
14. Marang L, Reiller PE, Eidner S, Kumke MU, Benedetti MF. Combining spectroscopic and potentiometric approaches to characterize competitive binding to humic substances. Environ Sci Technol. 2008;42(14):5094-8.
15. Vidali R, Remoundaki E, Tsezos M. An Experimental and Modelling Study of Cu2+ Binding on Humic Acids at Various Solution Conditions. Application of the NICA-Donnan Model. Water, Air, & Soil Pollution. 2010;218(1-4):487-97.
16. Xu J, Tan W, Xiong J, Wang M, Fang L, Koopal LK. Copper binding to soil fulvic and humic acids: NICA-Donnan modeling and conditional affinity spectra. J Colloid Interface Sci. 2016;473:141-51.
17. Wang P, Ding Y, Liang Y, Liu M, Lin X, Ye Q, et al. Linking molecular composition to proton and copper binding ability of fulvic acid: A theoretical modeling approach based on FT-ICR-MS analysis. Geochimica et Cosmochimica Acta. 2021;312:279-98.

18. Oste LA, Temminghoff EJ, Lexmond TM, Van Riemsdijk WH. Measuring and modeling zinc and cadmium binding by humic acid. Anal Chem. 2002;74(4):856-62.
19. Ratie G, Chrastny V, Guinoiseau D, Marsac R, Vankova Z, Komarek M. Cadmium Isotope Fractionation during Complexation with Humic Acid. Environ Sci Technol. 2021;55(11):7430-44.
20. Jouvin D, Louvat P, Juillot F, Marechal CN, Benedetti MF. Zinc isotopic fractionation: why organic matters. Environ Sci Technol. 2009;43(15):5747-54.
